# Identification of protein biomarkers associated with congenital diaphragmatic hernia in human amniotic fluid

**DOI:** 10.1038/s41598-023-42576-2

**Published:** 2023-09-19

**Authors:** Sumit Bhutada, Karin Tran-Lundmark, Benjamin Kramer, Peter Conner, Ashley M. Lowry, Eugene Blackstone, Bjorn Frenckner, Carmen Mesas-Burgos, Suneel S. Apte

**Affiliations:** 1 Department of Biomedical Engineering-ND20, Cleveland Clinic Lerner Research Institute, 9500 Euclid Avenue, Cleveland, OH 44195 USA; 2 Department of Experimental Medical Science and Wallenberg Center for Molecular Medicine, Lund University, Lund, Sweden; 3 The Pediatric Heart Center, Skane University Hospital, Lund, Sweden; 4 Department of Thoracic and Cardiovascular Surgery, Heart, Vascular, and Thoracic Institute, Cleveland Clinic, Cleveland, OH USA; 5 Department of Women’s and Children’s Health, Karolinska Institute, Stockholm, Sweden; 6 Department of Quantitative Health Sciences, Cleveland Clinic, Cleveland, OH USA

**Keywords:** Proteomics, Paediatric research

## Abstract

Congenital diaphragmatic hernia (CDH) is a severe birth defect frequently associated with pulmonary hypoplasia, pulmonary hypertension, and heart failure. Since amniotic fluid comprises proteins of both fetal and maternal origin, its analysis could provide insights on mechanisms underlying CDH and provide biomarkers for early diagnosis, severity of pulmonary changes and treatment response. The study objective was to identify proteomic changes in amniotic fluid consistently associated with CDH. Amniotic fluid was obtained at term (37–39 weeks) from women with normal pregnancies (n = 5) or carrying fetuses with CDH (n = 5). After immuno-depletion of the highest abundance proteins, off-line fractionation and high-resolution tandem mass spectrometry were performed and quantitative differences between the proteomes of the groups were determined. Of 1036 proteins identified, 218 were differentially abundant. Bioinformatics analysis showed significant changes in GP6 signaling, in the MSP–RON signaling in macrophages pathway and in networks associated with cardiovascular system development and function, connective tissue disorders and dermatological conditions. Differences in selected proteins, namely pulmonary surfactant protein B, osteopontin, kallikrein 5 and galectin-3 were validated by orthogonal testing using ELISA in larger cohorts and showed statistically significant differences aiding in the diagnosis and prediction of CDH. The findings provide potential tools for clinical management of CDH.

## Introduction

Congenital diaphragmatic hernia (CDH) results from incomplete diaphragm morphogenesis during fetal development. It is one of the most dreaded causes of severe respiratory failure in the neonate, requiring intensive long-term in-hospital care and is associated with the highest costs of all pediatric surgical conditions in the USA^1^. The incidence of CDH varies from 1.7 to 5.7 per 10,000 livebirths depending on the study population^2^. Comparison of the mortality rates of different congenital gastrointestinal anomalies found that CDH had the highest mortality of all, varying from 14.2% in high-income countries to 38.5% in middle-income countries and suggesting that the outcome may be far worse in low-income countries or when additional anomalies occur^3^. Although the etiology of CDH is multifactorial and possibly polygenic, the common end-result of an embryonic diaphragmatic defect is that it permits entry of abdominal viscera into the chest during the fetal period, impairing proper lung development. As a result, infants with CDH are born with varying degrees of pulmonary hypoplasia, delayed lung maturation, and alterations in pulmonary vascular structure, as well as other associated birth defects^4,5^. CDH can thus present a broad spectrum of severity, with pulmonary hypoplasia and pulmonary hypertension being major contributors to mortality and long-term morbidity of this disease. In recent years, ventricular dysfunction was described as an additional major contributor to CDH pathophysiology^6^. Better understanding of this pathogenic trifecta has led to improved survival rates during the past decades^1,7^, although its mechanisms are poorly understood. Advances in prenatal care, especially fetal imaging, have improved antenatal diagnosis of CDH and prediction of disease severity in a majority of affected fetuses^8–10^. This capability now provides an opportunity for better severity stratification and design of prenatal interventions to improve patient outcomes, but there are few molecular biomarkers available to facilitate this.

Amniotic fluid provides mechanical protection, nutrients and other molecules required for fetal growth and well-being and comprises both maternal serum-derived components and proteins of fetal origin^11^. Their relative contribution changes as gestation progresses, with a greater fetal contribution, especially from urine, occurring near the end of gestation^12^. For example, it is now known that increasing levels of amniotic fluid enzymes and electrolytes in late gestation correlate with the formation of the fetal kidneys, lungs and the gastrointestinal tract^11^. Thus, amniotic fluid composition potentially provides a window on fetal development and its anomalies. Liquid chromatography-tandem mass spectrometry, which provides high resolution and throughput, has been previously used to investigate amniotic fluid proteomes for insights on the physiology and complications of pregnancy as well as fetal anomalies^13–16^, although it has seen very limited use for human CDH. One prior study used proteomics of the amniotic fluid phospholipid profile in a sheep model of CDH treated with tracheal occlusion and identified specific changes responsive to the treatment^17^. A comparison of CDH and normal amniotic fluids using NMR spectroscopy identified spectral differences between the two groups which suggested that specific differences in metabolites may occur in CDH^18^. However, a broad discovery-oriented comparison of amniotic fluid proteomes in human CDH remains to be undertaken.

Like other body fluids, proteomic analysis of amniotic fluid is challenging due to its high protein dynamic range, which can limit identification of low abundance components. Here, we utilized a high-resolution quantitative proteomics strategy for analysis of amniotic fluid collected at term from women with healthy and CDH pregnancy and addressed the challenge of proteome complexity by immunodepletion of the most abundant components. We subsequently investigated the findings of this discovery-oriented proteomics approach orthogonally using ELISA of selected components in a larger number of samples. These included the gestational term samples used for proteomics, where the findings were validated, as well as others obtained at earlier gestational time points to determine if the observed differences could be replicated and to consider whether the findings would be broadly applicable as CDH biomarkers.

## Results

### Global protein profiling of amniotic fluid in CDH and control discovery groups

Amniotic fluid samples were collected and analyzed quantitatively from women with healthy fetuses (n = 5) and fetuses with CDH (n = 5) at term. To facilitate detection of less abundant proteins by LC–MS/MS, 14 highly abundant proteins were first depleted from 1 mg of total protein from each sample (exemplified by the chromatographic profile in Figure S1). This enrichment for low abundance proteins increased overall protein identification by approximately fivefold (data not shown), with detection of 1036 high confidence proteins overall (Supplemental Excel file), although 5 highly abundant proteins—serum albumin (ALB), isoform 3 of vitamin D-binding protein (GC), serotransferrin (TF), hemopexin (HPX), and ceruloplasmin (CP) still comprised 25% of the total ion score. Pearson correlation analyses (Figure S2) revealed a minimum inter-cohort R value of 0.88. Samples within the cohorts had a higher correlation, with a minimum R value of 0.91.

### Comparison of the amniotic fluid proteome from pregnancies with and without CDH reveals inter-cohort differences

**Figure 1:**
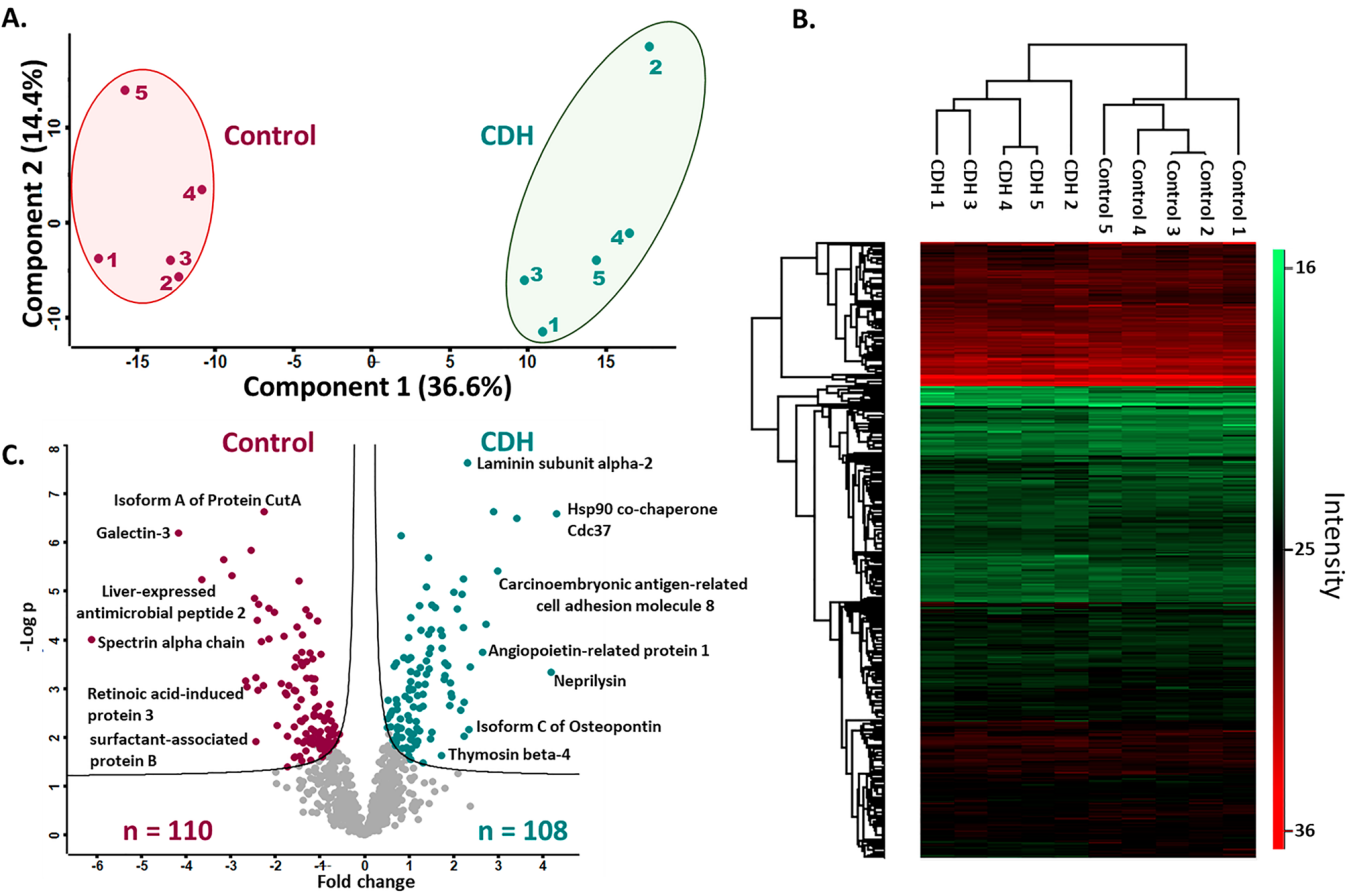
Quantitative proteome changes in amniotic fluid from pregnancies with CDH (n = 5) and healthy pregnancies (n = 5). (**A**) Principal component analysis based on all identified proteins shows that the first and second component segregate the control and CDH groups and account for 36.6% and 14.4% of the variability respectively. (**B**) Heat map of z-scored protein abundances (LFQ intensities) after unsupervised hierarchical clustering with FDR < 0.05. The heat map was generated using Perseus V1.6.14.0. (https://maxquant.net/perseus/). (**C**) Volcano plot of the p values vs. the log10 protein abundance differences of all identified proteins. The colored circles show proteins with statistically significant altered abundance in the respective cohorts.

To ascertain whether overall similarities and differences between the control and CDH cohorts could be uncovered by proteomics, principal component analysis (PCA) of the data was employed and indicated distinct segregation of the amniotic fluid cohorts (Fig. 1A). For functional/quantitative changes in protein abundance between the cohorts, unsupervised hierarchical clustering with FDR < 0.05 was also performed. Similar to PCA, clustering of samples by cohort was observed in heat maps (Fig. 1B). Statistical analysis yielded 218 differentially abundant proteins with *p*-value < 0.05, with 110 proteins having higher abundance in controls and 108 proteins with higher abundance in the CDH cohort; 25 proteins were identified in only one cohort or the other, but in all the samples in that cohort (Fig. 1C; Tables S2–S4). These proteins, constituting candidate biomarkers, explained the broad inter-cohort distinctions identified by PCA and unsupervised hierarchical clustering.

All identified proteins were annotated for their subcellular localization based on gene ontology. A majority of the proteins were of extracellular origin (41%), followed by cytoplasm (31%) and plasma membrane (22%) origins (Figure S3A), which correlated well with the amniotic fluid proteome as previously described^19,20^. The CDH amniotic fluid had a higher representation of secreted (extracellular) and plasma membrane proteins compared to the controls, which had a greater proportion of cytosolic proteins (Figure S3A). We categorized proteins based on biological process and molecular function using PANTHER database version 7.1 (www.pantherdb.org). Based on their molecular functions, proteins with higher abundance in CDH may be classified into six groups: translation regulator activity (2%), molecular transducer activity (9%), binding (43%), molecular function regulator (4%), catalytic activity (35%) and transporter activity (7%) (Figure S3B). Although similar categories were identified in both CDH and control the control amniotic fluid had a markedly higher proportion assigned as molecular function regulator and a reduction in the molecular transducer activity group. Proteins with higher abundance in CDH were classified into thirteen groups of biological processes with proteins annotated as belonging to developmental processes higher in control amniotic fluid.

### Pathway analysis and network construction shows activation of the GP6 signaling pathway in CDH

**Figure 2:**
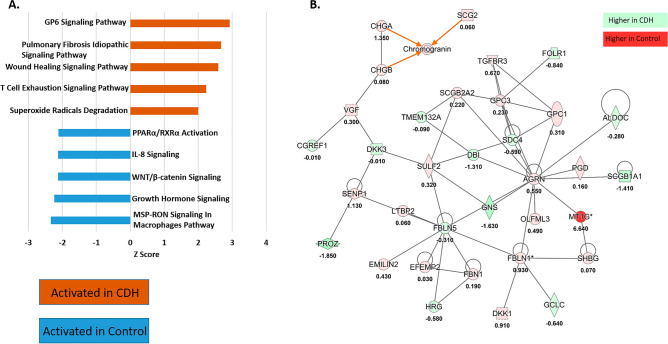
Canonical pathways and networks affected in the CDH proteome. (**A**) Bar graph representing the top 5 canonical pathways activated in CDH and control amniotic fluid at term. (**B**) The top network mapped by IPA was associated with “cardiovascular system development and function, connective tissue disorders, dermatological diseases and conditions”.

Possible CDH-associated canonical pathways were sought using the IPA core analysis module and overlaid with the Ingenuity Knowledge Base annotation. The top 5 canonical pathways based on their z-score from the expression data showed that the GP6 signaling pathway was the most upregulated in CDH and the MSP–RON signaling in macrophages pathway was the most upregulated in controls (Fig. 2A). A total of 25 networks were constructed by IPA and the one with the highest score is shown in Fig. 2B. This particular network shows 34 gene products with different levels in CDH and control amniotic fluids that work together in cardiovascular system development and function, connective tissue disorders and dermatological diseases and conditions; all proteins associated with this network were identified in our dataset and their expression ratios are overlaid.

**Figure 3:**
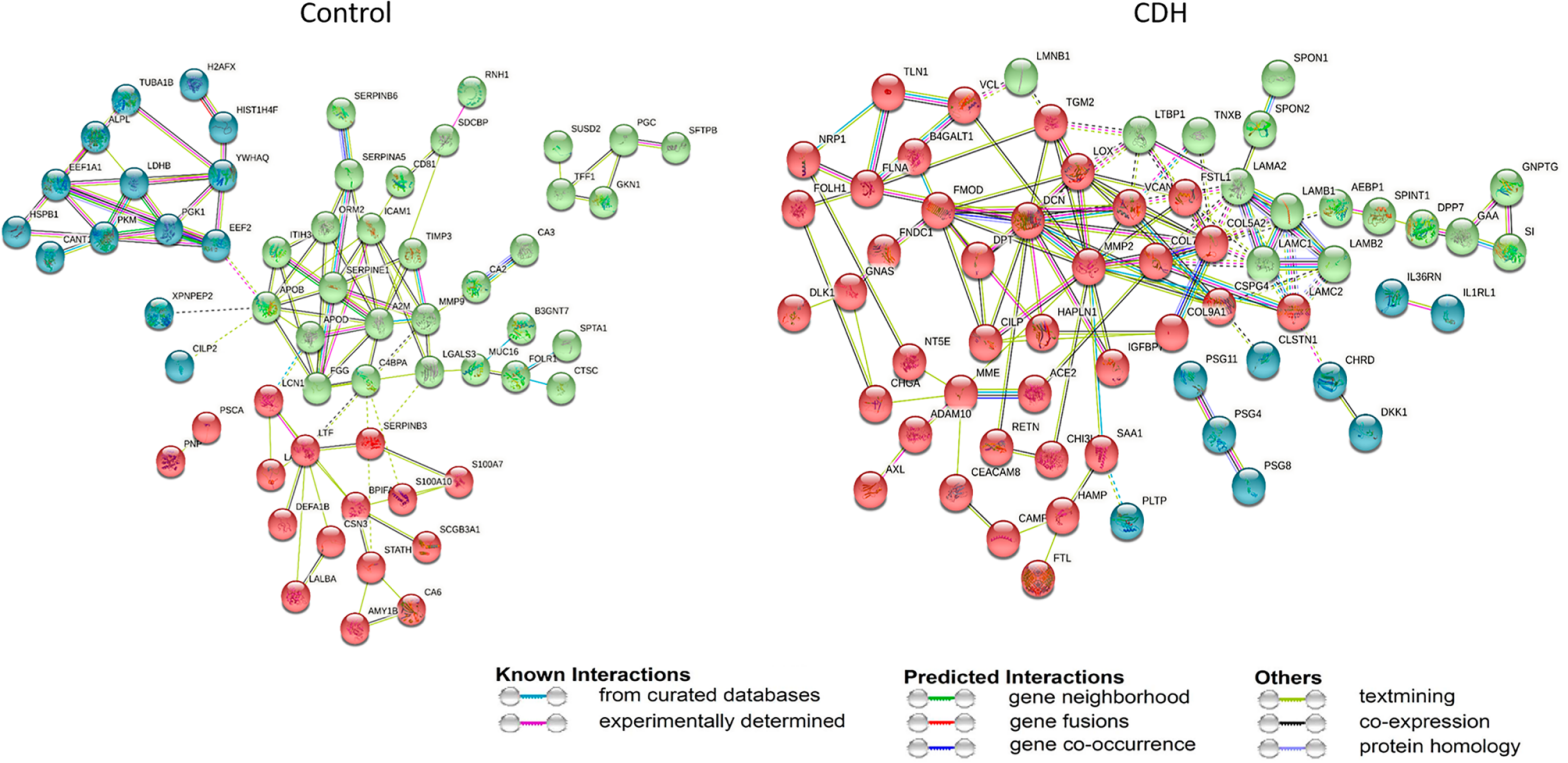
Protein–protein network analysis using the STRING database. K-means clustering was applied and the top networks in control and CDH amniotic fluid are represented. The edges indicate both functional and physical protein associations. Thicker lines show stronger intermolecular connections.

For possible functional associations of protein changes in CDH vs control amniotic fluid, we searched the available protein–protein interactions of the quantified proteins in the STRING database, which contains information on known and predicted, direct physical and indirect functional protein–protein interactions. K-means clustering was applied to determine the three most prominent biological processing clusters. Proteins that were more abundant in CDH shown in cluster 1 (red) were defined by 38 connecting nodes and found to be enriched for the dermatan sulfate biosynthetic process; cluster 2 (green) was defined by 15 nodes and was enriched for extracellular matrix organization and cluster 3 (blue) had 9 nodes and was enriched for positive regulation of cardiac muscle contractions. Proteins with higher abundance in the control group showed cluster 1 (red) to be enriched for negative regulation of smooth muscle cell matrix adhesion (16 nodes), cluster 2 (green) to be enriched for antibacterial humoral response (29 nodes) and cluster 3 (blue) was enriched for ficolin-1-rich granule lumen (14 nodes) (Fig. 3). The independent nodes were removed since they were not part of any network.

### ELISA validation of selected differentially abundant proteins identified in proteomic analysis

Quantitative ELISA in a total of 40 amniotic fluid samples, including the 10 samples initially used for proteomics was performed for orthogonal analysis of four proteins selected from among the 25 proteins with absolute difference values among the 218 proteins with statistically significant differential abundance in control and CDH amniotic fluid. These proteins (pulmonary surfactant protein B (SP-B), osteopontin, kallikrein-5 and galectin-3) were selected primarily on the basis of potential mechanistic and clinical relevance to the lung or pulmonary hypertension (SP-B, osteopontin, kallikrein-5) and availability of high-quality ELISA assays, yet a paucity of information regarding their role in CDH. 

**Figure 4:**
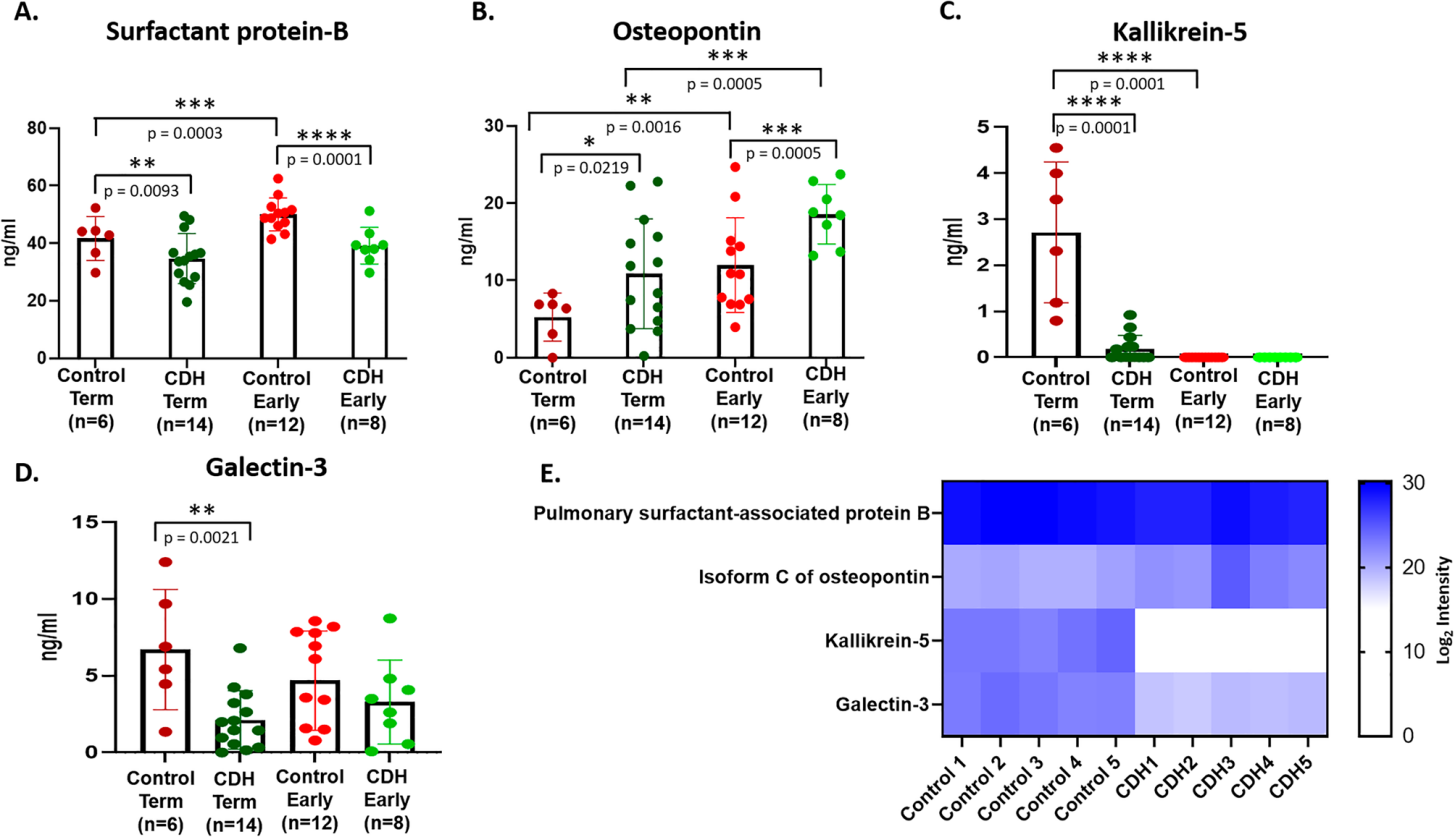
Quantitative ELISA of selected proteins. 40 amniotic fluid samples collected both during the second trimester (early) and at term gestation from control and CDH pregnancies were analyzed and ELISA was performed in technical duplicates to provide assurance of reproducibility. All duplicate assays showed similar outcomes; an unpaired t test was applied between the two groups to determine significant changes and* p* < 0.05 was considered to be significant. The following were analyzed: (**A**) Pulmonary surfactant-associated protein B. (**B**) Osteopontin. (**C**) Kallikrein 5, and (**D**) Galectin-3. (**E**) A heat map for these four selected proteins was generated using log 2 transformed intensities from the amniotic fluid label-free proteomics dataset and appeared to be consistent with the ELISA results. The heat map was generated using GraphPad Prism 9 (https://www.graphpad.com/).

SP-B is an essential lipid-associated protein that imparts surfactant-like properties to the alveolar surface^21^. 52 SP-B peptides were identified with 67% coverage of its protein sequence. SP-B was identified in all 10 samples and was more abundant in the control cohort than the CDH cohort at term (Fig. 4E). SP-B ELISA confirmed statistically significant higher abundance of SP-B in control cohorts than CDH both during early gestation and at term (Fig. 4A). Although SP-B levels were higher during the second trimester than at term in controls, the CDH group did not show this temporal difference (Fig. 4A). Amniotic fluid samples collected both during the second trimester and at term from 4 women with CDH affected children, showed no statistical differences in SP-B levels at the two time points (p value = 0.7260) (Figure S4).

Osteopontin was identified by 92 peptides that provided ~ 61% sequence coverage. The multiple isoforms of osteopontin have largely identical sequences other than specific alternatively spliced exons. In addition to peptides from regions shared by all isoforms, isoform c (UniProt ID P10451-3) was identified by a single high-confidence peptide spanning the specific splice junction (Figure S5) and a statistically significant increase was detected in CDH amniotic fluid (Fig. 1C). Since a specific ELISA for isoform c is unavailable, an ELISA that detects all isoforms was used and revealed a statistically significant increase in osteopontin abundance in CDH vs controls both during early gestation and at term (Fig. 4B). Amniotic fluid samples collected both during the second trimester and at term from 4 women with CDH affected children showed higher osteopontin levels during the first trimester which appeared to be reduced at term, although the difference was not statistically significant (Figure S4), likely due to the small sample size.

Kallikrein 5**,** a serine protease which is highly expressed in fetal and adult epidermis, was identified by proteomics in all 5 control samples, but none of the CDH samples, with 3 high confidence peptides providing 14% sequence coverage. ELISA revealed that kallikrein-5 was below detection limits during the second trimester, but was detectable at term in control samples, although not CDH (Fig. 4C). Amniotic fluid collected longitudinally from 4 women with CDH during the second trimester and at term showed no expression of kallikrein-5 either during the second trimester or at term except for one sample (Figure S4).

Galectin-3 is a carbohydrate binding protein that participates in lattices on the plasma membrane where it may influence the expression, localization, and activity of several cell surface receptors and modulate cellular functions such as signaling, migration, and adhesion^22^. Galectin-3 was identified by 3 high confidence peptides providing 8% sequence coverage. Galectin-3 ELISA showed a statistically significant reduction in CDH samples at term (Fig. 4D), consistent with the proteomics analysis (Fig. 4E), but no difference was observed during early gestation. Samples collected longitudinally from 4 pregnancies with CDH showed galectin-3 to be higher in the second trimester than at term except for one outlier (Figure S4). Since the outliers in the galectin-3 and kallikrein 5 analysis arose from different samples, no particular sample was identified as an outlier and no samples were excluded in the ELISA analysis.

### Prediction model of CDH based on ELISA assays

**Figure 5:**
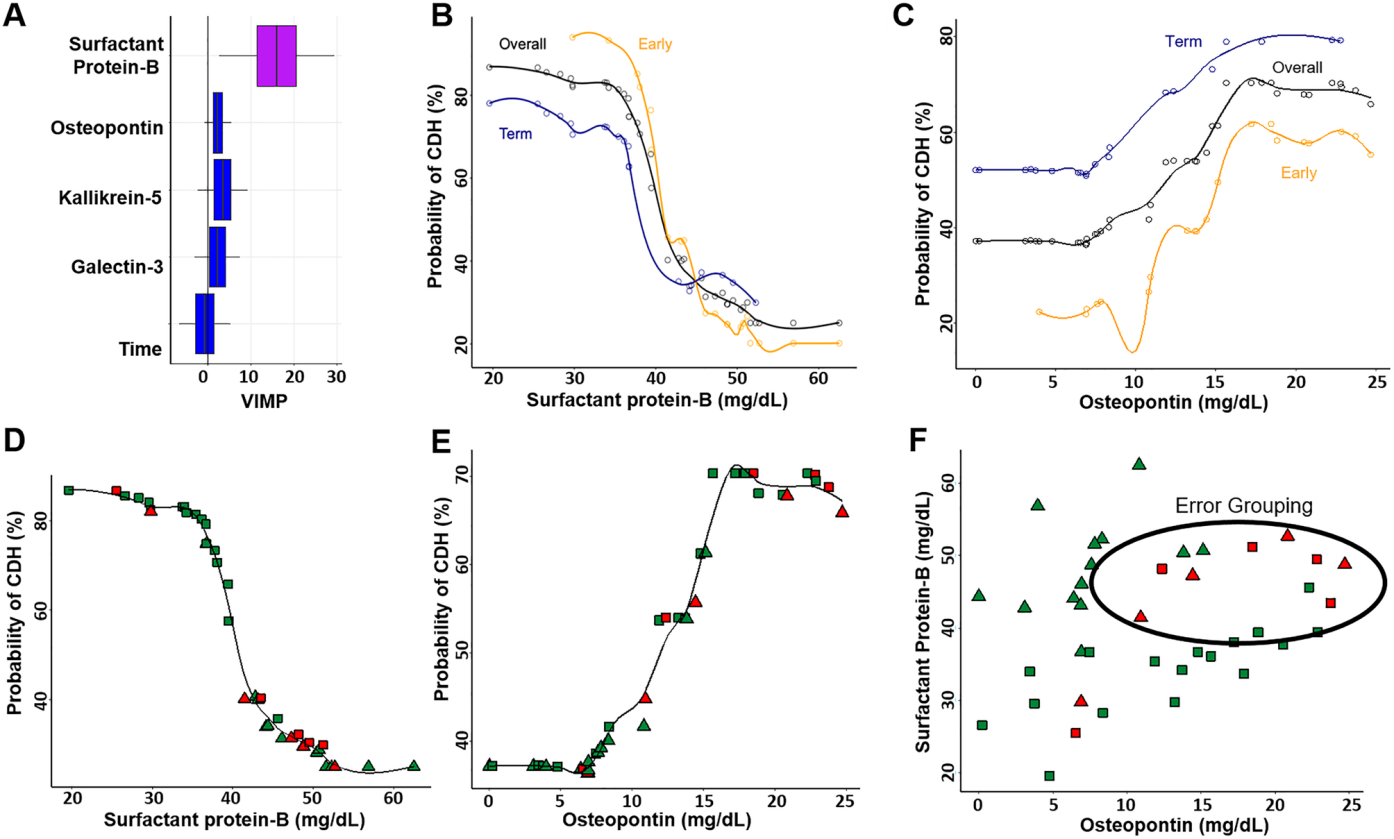
Random Forest Prediction Model demonstrating contributions of 4 ELISA protein concentrations and time of amniotic fluid sampling on the prediction of CDH. (**A**) Variable of importance (VIMP) plot. Variables with larger values are more important to the random forest model in predicting CDH. Boxplots present 95% confidence intervals (95% C.I.) of covariates’ contribution to the model. Covariates that contributed significantly, 95% C.I. did not include zero, are filled in purple and those with 95% C.I. included zero are blue. (**B**) Partial-dependence adjusted graph of association between SP-B and predicted likelihood of CDH, stratified by time of amniotic fluid sampling (*Yellow—*Early, *Navy—*Term). Adjusted CDH probability is based on random forest classification. Individual patients are denoted by the unfilled circles. (**C**) Partial-dependence adjusted graph of association between osteopontin and predicted likelihood of CDH, stratified by time of amniotic fluid sampling (*Yellow—*Early, *Navy—*Term). Adjusted CDH probability is based on random forest classification. (**D**) Unstratified partial-dependence adjusted graph of association between SP-B and predicted likelihood of CDH with model error detection labels. CDH population (squares) and healthy controls (triangles) are filled by model grouping assignment. Green indicates when the group is correctly categorized by the model, and red when incorrectly categorized. (**E**) Unstratified partial dependence-adjusted graph of association between osteopontin and predicted likelihood of CDH with model error detection labels. CDH population (squares) and healthy controls (triangles) are filled by model grouping assignment, green when the group is correctly categorized by the model, and red when incorrectly categorized. (**F**) Scatter plot of patients based on SP-B and osteopontin concentrations with model error detection labels. CDH population (squares) and healthy controls (triangles) are filled by model grouping assignment, green when the group is correctly categorized by the model, and red when incorrectly categorized.

Random forest is a nonparametric statistical ensemble method which makes no distributional, functional (linear or nonlinear), or interaction effect assumptions about covariate relationships to predict the response^23^). Initial evaluations using traditional logistic regression analyses (Supplemental methods, Table S5-S6, Figure S6) violated necessary assumptions of linearity, encouraging the more robust random forest approach. SP-B, osteopontin, kallikrein-5, galectin-3 levels from each cohort, and the time points (early vs. term) of amniotic fluid collection were utilized to develop a prediction model, using random forest to assess the probability of CDH (Supplemental methods). Our model demonstrated that SP-B was the most influential predictor of CDH, with a variable importance (VIMP) value of 16.1 (95% C.I. 2.7–29.4), and that gestational age at amniotic fluid collection least improved the model, VIMP value − 0.6 (95% C.I. − 6.7–5.4, Fig. 5A). We then evaluated the independent effect of each protein on the probability of CDH. This demonstrated that SP-B had a nonlinear relationship with predicting the probability of CDH, with a transition point of approximately 36 mg/dL, independent of when the amniotic fluid was collected (Fig. 5B). A similar non-linear relationship was observed with osteopontin, although it did not reach statistical significance (Fig. 5C). The overall out of bag error of the prediction model was 0.22. Individuals were most commonly misclassified as either CDH or control when the opposite was true, when both SP-B (Fig. 5D) and osteopontin (Fig. 5E) concentrations were elevated (Fig. 5F).

## Discussion

The present study initially sought global proteomic changes in amniotic fluid from pregnancies without detected anomalies and those with CDH and observed significant differences in abundance of several proteins between the two cohorts. To overcome the inherent challenge posed by high abundance proteins in amniotic fluid and to extend proteome coverage sufficiently to include low abundance proteins, all samples were first subjected to an antibody-based affinity-isolation method to reduce levels of 14 highly abundant proteins. This step, combined with extensive fractionation prior to proteomics analysis of the resulting fractions on a high-resolution mass spectrometer led to an eventual total yield of 1036 proteins. From among the consistently observed changes in the amniotic fluid proteome, 4 proteins were selected for further analysis, of which 3 were chosen for their relevance to the principal disease processes thought to be clinically significant in CDH (i.e., pulmonary immaturity and pulmonary hypertension).

One of the key pathologic events that characterizes CDH is under-development of the lungs, therefore changes in the surfactant protein content in amniotic fluid may be a helpful indicator of lung maturity. Surfactant analysis (including phosphatidylcholine and the four-surfactant proteins, one being SP-B) in infants and animal models of CDH was inconclusive in part because of differences in the samples related to age, location and analytical method. After measurement of SP-A and saturated phosphatidylcholine in amniotic fluid Moya et al. concluded that reduced surfactant was a characteristic of CDH, but the study did not measure SP-B^24^. Another study which concluded that there was no difference in amniotic fluid levels of surfactant also did not measure SP-B^25^. In a surgical rabbit model of CDH, SP-B gene expression was reduced compared to the sham controls, as was the SP-B staining intensity determined by immunohistochemistry^26^. Boucherat et al. compared surfactant levels in fetuses with CDH with unaffected fetuses at different time points during pregnancy and observed that affected lungs were significantly smaller and histologically immature, but no delay in surfactant development or content (including SP-B) in CDH fetal lungs was observed by western blotting^27^. Although surfactant di-saturated phosphatidylcholine (DSPC) synthesis and surfactant-A were not different in tracheal aspirates taken from infants with or without CDH^28^, a subsequent study from the same group found reduced SP-B fractional synthesis rate and amount present in tracheal aspirates from infants with CDH compared to infants without lung disease^29^. The present work suggests a strong association between CDH and reduced SP-B in amniotic fluid, where it was not previously directly evaluated by proteomics in the context of CDH. Support for the association, moreover, was independently obtained by ELISA and identified as influential in the prediction of CDH. It should be noted that our observation of reduced SP-B in CDH amniotic fluid does not purport to resolve the controversy of whether CDH is associated with a developmental delay in surfactant expression, since reduced surfactant in amniotic fluid could simply reflect smaller lungs^27^.

Osteopontin, which our LC–MS/MS analysis identified at higher levels in CDH, is highly expressed by pulmonary artery smooth muscle cells and adventitial fibroblasts from patients with pulmonary hypertension^30,31^ and osteopontin levels in serum correlate with pulmonary hypertension severity^32,33^, prompting us to select it for further evaluation. The random forest model suggested that osteopontin was the second most influential variable in predicting CDH, although it did not reach statistical significance, which may change with a larger sample size. There are three spliced isoforms of the *OPN* gene in humans, (a) OPN a—full-length protein with a molecular weight of ∼54 kDa, which is the most prevalent and regarded as the canonical isoform 9b) OPN b, which lacks exon 5 and has a molecular weight of ∼50 kDa and (c) OPN c which lacks exon 4, with a molecular weight of ∼47 kDa. The regulation and exact function of each isoform is still unclear, although isoform c is reported to be upregulated in ovarian, breast and pancreatic cancer and suggested to promote cell survival under drug-induced cytotoxic pressure^34^. The observed changes in isoform c in amniotic fluid require future consideration, since an isoform-specific antibody or ELISA is presently unavailable.

Kallikreins are secreted serine proteases abundant in various body fluids such as blood, seminal plasma, sweat, saliva, cerebrospinal fluid, milk, interstitial spaces and cervico-vaginal fluid and they can activate or inactivate many proteases, suggesting a broad impact^35^. Kallikrein 5 is most abundant in the skin and is involved in skin desquamation which may explain its presence in the amniotic fluid^36^. However, there is no prior suggestion of an association with CDH or pulmonary hypertension. Galectin-3 was reported as a biomarker in pulmonary hypertension and associated right ventricular dysfunction and has been shown to stimulate the proliferation of smooth muscle cells in vitro^32,37–39^. It was reported to be elevated in amniotic fluid following cytomegalovirus infection^40^, but an association with CDH was not previously reported.

In summary, our findings, made by initially applying quantitative global protein mass spectrometry to amniotic fluids obtained from normal or CDH pregnancies suggested differences in composition, which may provide novel biomarkers for improved diagnosis, insights on pathogenesis and potentially help in evaluating the response to treatment. Currently, there is no molecular panel for detection of CDH and guidance of clinical management, which this analysis may help to develop, especially if the changes are reflected in maternal serum or urine. SP-B, osteopontin and galectin-3 have an established association with pulmonary disease and may be useful biomarkers to assess the degree of pulmonary hypoplasia and pulmonary hypertension in CDH and monitor the efficacy of treatment aiming to restore normal lung airway and vascular physiology in CDH. The differences in levels of an additional 214 differentially abundant proteins remain unconfirmed at the present time, but it is possible that a panel of useful biomarkers could be developed from the present findings. This could be done by a mass-spectrometry-based parallel reaction monitoring assay, data independent analysis of the amniotic fluids or antibody and aptamer-based methods that assemble a panel containing all 218 proteins for further evaluation, with the four already studied here providing a benchmark. The findings can have important implications for better risk stratification and design of effective therapeutic interventions for CDH, potentially based on the proteome difference identified here.

### Limitations of the study

1. The sample size for global proteomics analysis using label-free quantitation was necessarily modest with five samples in each cohort and was limited by the protein depletion and fractionation steps. In-depth proteomics analysis was undertaken primarily as a first-level discovery strategy, and a limited number of selected changes were validated orthogonally by ELISA using a larger sample number.
2. The present analysis used a label-free approach in which samples are not all run together simultaneously on the mass spectrometer. Accordingly, 1. We performed replicate runs a month apart to minimize instrument-dependent variables and 2. Selected proteins were validated using ELISA in a greater number of samples than employed for the initial proteomics analysis.
3. Immunodepletion of high abundance proteins as an additional experimental step may lead to sample loss and loss of proteins which bind the depleted ones. Nevertheless, depletion did provide a five-fold increase in the number of proteins identified post-depletion.
4. Since CDH is likely to have a multifactorial origin, it is likely that there is considerable variation in primary etiology, severity, and outcomes in the study CDH cohort. Our prediction model is unable to infer the physiologic relevance of the selected biomarkers and offers only a snapshot based on the time of sample collection. No model is presented to account for the multifactorial origin and development of CDH. Nevertheless, both the strong relationships between SP-B and osteopontin in our model and the clear segregation of cohorts in PCA suggests that consistent proteomic changes occur in amniotic fluid in CDH despite the likely heterogeneous etiopathogenesis.

### Conclusions

Although CDH is a complex condition with potentially many underlying causes, comparative quantitative proteome analysis of amniotic fluid from pregnancies with and without CDH suggests that a large cross-section of the proteome is consistently affected by CDH. Orthogonal quantitation of four components with statistically significant differences between CDH and normal cohorts validated their observed differences in proteomics and aided in the development of a prediction model of CDH. These and other differentially abundant amniotic fluid components may provide prognostic value for clinical detection and evaluation of CDH, and support future investigations into their association with disease severity and treatment response.

## Materials and methods

### Study participants

Amniotic fluid was obtained by amniocentesis under ultrasound after informed written consent for collection and use of samples from all study participants. All research was conducted with the approval of the regional ethical committee in Stockholm, Sweden (Dnr 2012/2037–31/4) in accordance with relevant guidelines/regulations and in accordance with the Declaration of Helsinki. Participants were recruited at a single tertiary center for fetal medicine and postnatal CDH management (Karolinska Institute, Stockholm, Sweden) between 2013-03-01–2017-12-31. Amniotic fluids (10 ml) were obtained from women with healthy singleton pregnancies between 15 and 20 gestational weeks (gw) (control cohort), women carrying fetuses with CDH obtained at the time of diagnosis (between 18 and 21 gw, CDH cohort) and at term (between 37 and 39 gw, for controls and CDH). The inclusion criteria were as follows: singleton pregnancy without any structural malformations at the time of the second trimester scan (controls) or singleton pregnancy with diagnosis of isolated CDH (CDH). We excluded participants with associated chromosomal or other structural abnormalities. Relevant characteristics of the cohorts are summarized in Table S1. Amniotic fluids were centrifuged at 1500 g at 4 °C for 20 min to remove cells and debris and stored at − 80 °C.

### Depletion of high-abundance proteins

Thawed amniotic fluids were again centrifuged at 1500 g at 4 °C for 20 min. The supernatant was transferred to a new tube and Halt protease inhibitor cocktail (catalog number 78430, ThermoFisher Scientific) was added. 2–3 ml amniotic fluid was concentrated to 100 µl using Amicon ultra 0.5 ml, 3 kDa molecular weight cut-off columns (catalog number UFC501024, Millipore). The total protein content was determined by the Bradford assay (catalog number 5000006, Bio-Rad) and a volume equivalent to 1 mg of total protein from each sample was subjected to immunodepletion of 14 highly abundant serum proteins (albumin, IgG, α1-antitrypsin, IgA, IgM, transferrin, haptoglobin, α2-macroglobulin, fibrinogen, complement C3, α1-acid glycoprotein (orosomucoid), HDL (apolipoproteins A-I and A-II), LDL (mainly apolipoprotein B)) with a Seppro-Supermix LC5 column (catalog number SEP060, Millipore-Sigma) attached to a Waters HPLC system (Milford, MA). Unbound and depleted fractions were collected between 5 and 25 min with a flow rate of 500 µl/ min (Figure S1). The depleted fractions (unbound) were concentrated using molecular weight cut-off columns and buffer-exchanged to 50 mM ammonium bicarbonate, pH 7.8. Total protein content post-depletion was measured using the Bradford assay.

### In-solution trypsin digestion

50 µg of total protein from each sample was reduced with 5 mM dithiothreitol DTT (final concentration) at 60 °C for 1 h, followed by alkylation with 15 mM iodoacetamide (IAA, final concentration) for 30 min in the dark at room temperature. The reaction was diluted with three volumes of 50 mM ammonium bicarbonate (pH 7.8) and trypsin (catalog number V5280, Promega) was added in a 1:50 (trypsin: protein) ratio and incubated overnight at 37 °C. Digest clean-up was done using C18 columns (Sep-Pak, catalog number WAT023590, Waters) and peptides were eluted in 500 µl of elution buffer (25% water + 75% acetonitrile + 0.1% trifluoracetic acid), dried using a SpeedVac (ThermoScientific Savant SPD1030-230) and reconstituted in 50 µl of 1% acetic acid. 5 µl of this peptide mixture was injected into the mass spectrometer for analysis.

### LC–MS/MS

Tryptic digests were analyzed in technical duplicates with a gap of one month between analyses using a Thermo Ultimate 3000 UHPLC interfaced with a ThermoFisher Scientific Fusion Lumos tribrid mass spectrometer system (Thermo Fisher, MA, USA). The HPLC column was a Dionex 15 cm × 75 µm id Acclaim Pepmap C18, 2 μm, 100 Å reversed phase capillary chromatography column. The peptides were eluted from the column using solvent A (0.1% formic acid in water) and solvent B (0.1% formic acid in acetonitrile). LC gradient parameters were as follows and shown as time (% of B): 0 min (2%), 5 min (2%), 85 min (35%), 100 min (90%), 101 min (2%) and 111 min (2%) at a flow rate of 0.3 μL/min, and peptides were introduced into the source of the mass spectrometer on-line.

The nanospray ion source was operated at 1.9 kV. The digest was analyzed using a data-dependent method with 35% collision-induced dissociation fragmentation of the most abundant peptides every 3 s and an isolation window of 0.7 m/z for ion-trap MS/MS. Scans were conducted at a maximum resolution of 120,000 for full MS. Dynamic exclusion was enabled with a repeat count of 1 and ions within 10 ppm of the fragmented mass were excluded for 60 s.

### Data analysis

RAW files with spectral information were analyzed using Proteome Discoverer 2.3, using the built-in SEQUEST search engine and searched against a human database downloaded from Uniprot (release 2018_11; UP000005640_9606) containing 26,037 sequences. Trypsin was used as the protein-cleaving enzyme and up to two missed cleavages were allowed. Parameters for protein identification were set as: precursor mass tolerance of 10 ppm, fragment mass tolerance of 0.6 Da, static modifications included carbamidomethyl (C), whereas dynamic modifications included oxidation (M). For high confidence assignment of peptides and protein identities, a 0.01 false discovery rate (FDR) for this analysis was applied by searching against a decoy library^41^. Proteins were included only if they were identified by a minimum of 2 high-confidence peptides. Label-free quantification was done using precursor ions quantifier node in Proteome Discoverer 2.3 and only unique and razor peptides were used for quantification.

### Pathway analysis

For Ingenuity Pathway Analysis (IPA) analysis, the SwissProt accession numbers along with the abundance ratios were uploaded to IPA software (Ingenuity Systems, Mountain View, CA). The proteins were mapped to disease and function categories and canonical pathways available in Ingenuity database that were ranked by the z-score.

### String analysis

A protein interactome map was used to predict protein networks including both physical interactions from experimental data and functional associations from curated pathways, automatic text mining, and prediction methods (https://string-db.org/).

### ELISA for pulmonary surfactant protein B, osteopontin, kallikrein 5 and galectin-3

ELISA kits (Human galectin-3 Quantikine ELISA Kit- catalog number DGAL30, human osteopontin (OPN) Quantikine ELISA Kit—catalog number DOST00; human Kallikrein 5 Quantikine ELISA Kit—catalog number DKK500; human SP-B/surfactant protein B ELISA Kit—catalog number NBP2-76690) were purchased from R&D systems (Bio-Techne, Minneapolis, MN, USA). Appropriate sample dilutions were performed. A standard curve was generated for each kit and the amniotic fluid samples were analyzed in duplicate (several weeks apart) following the manufacturer’s instructions.

### Statistical analysis

For label-free mass spectrometry data analysis, normalized precursor intensity values from Proteome Discoverer 2.3 were imported in Perseus V1.6.14.0. Intensity values were log-transformed. Proteins were filtered based on valid values and were only included if they were identified in 7 out of 10 samples. Missing values were imputed based on normal distribution. Abundance of proteins in the CDH and control groups was compared using a t-test with a 0.05 FDR correction applied for this specific analysis followed by determination of –Log(P-values)^42,43^.

Statistical analyses for ELISA were performed separately for individual assays using GraphPad Prism 9.4.0. Briefly optical density readings were imported in duplicates for each sample group and ordinary one-way ANOVA was applied to compare the groups. Significant difference was based upon *p* < 0.05.

Machine learning was implemented using R software 4.2.2. Categorical variables are summarized as frequencies and percentages; comparisons were made using chi-squared test or Fisher’s exact test when fewer than 5 events were observed in either group. Continuous variables are summarized as mean (standard deviation) and comparison were made using a pooled t-test, with significance determined by *p* values < 0.05. Data were re-analyzed using a random forest (RF) approach, to identify predictors of CDH based on ELISA results using the four selected proteins and the time of amniotic fluid collection. Implementing a forest of 1000 classification trees from a subset of 5 selected variables at each time point, the probability of CDH was determined^44^. Random forest variable importance (VIMP) values were used to hierarchically order covariates in relation to predicted CDH probability, with higher VIMP values reflecting more influential covariates^45^. A subsampling approach was utilized to determine the model-based prediction error and determine 95% confidence intervals (95% C.I.) for each variable^46^. Partial dependency plots were used to describe the relationship between covariates of interest and the response by risk-adjusting for all other covariates^47^. Additional details are provided in the supplement.

### Supplementary Information

Supplementary Information 1.

Supplementary Information 2.

Supplementary Information 3.

## Data Availability

The mass spectrometry proteomics data have been deposited to the ProteomeXchange Consortium via the PRIDE^48^ partner repository with the dataset identifier PXD041165 and 10.6019/PXD041165.
