# Supplementary material for: Identification of protein biomarkers associated with congenital diaphragmatic hernia in human amniotic fluid
Source: Sci Rep. 2023 Sep 19;13:15483. doi: 10.1038/s41598-023-42576-2 (PMC10509251; doi:10.1038/s41598-023-42576-2)
Supplement: Supplementary file 1 — Supplementary Information 1. [file 41598_2023_42576_MOESM1_ESM.docx]

**Identification of protein biomarkers associated with congenital diaphragmatic hernia in human amniotic fluid**

Sumit Bhutada, Karin Tran-Lundmark, Benjamin Kramer, Peter Conner, Ashley M. Lowry, Eugene Blackstone, Bjorn Frenckner_,_ Carmen Mesas-Burgos, and Suneel Apte

**Manuscript supplement**

The supplement contains:

Supplemental statistical methods

Tables S1-S4

Figures S1-S6

Supplemental data files (Excel (proteomics) and HTML (code) file)

**Random Forest**

Random Forests (RF) is a nonparametric statistical ensemble method that utilizes all the variables and makes no distributional or functional (linear or nonlinear) or interaction effects assumptions about covariate relationships to the response [1]. RF is a robust, nonlinear technique that optimizes predictive accuracy by fitting an ensemble of trees to stabilize model estimates. Each tree is created with a random subgroup of patients and each branch split is generated from a random subset of variables. Prognostic risk factors are selected by inspection of the forest for variables most frequently chosen to predict the response in each tree. It is most similar to classification and regression trees (CART) [2], but more robust because it utilizes a forest to analyze the data rather than a single tree. Response estimates are derived by aggregation across the individual tree estimates within the forest. RF is implemented using the *rfsrc* R-Package [3, 4].

RF-Classification (RF-C) [1] was used to analyze the categorical response (binary, ordinal, nominal). Each branch (node) split was optimally selected from a subset of 3 randomly selected candidate variables (by default, squared root of p variables randomly selected as candidate variables for splitting a node, where p is the number of variables, rounded to the nearest whole variable). Splitting a branch is carried out by using Gini index splitting rule [2]. For this RF-C analysis, a forest was grown using 1000 regression trees for presence of congenital diaphragmatic hernia (CDH) in the mother’s child using the selected ELISA assays and the time of blood sampling — 5 in all.

The RF variable importance (VIMP) measure was used to hierarchically order the variables in relation to a predicted response [5]. VIMP is calculated as the difference between the model-based prediction error of the response variable when the explanatory variable is noised-up and the model-based prediction error of response without any noising. If the explanatory variable is informative, then noising the variable results in much higher prediction error which in turn results in higher VIMP value. On the other hand, noising a non-informative explanatory variable result in a prediction error similar to the prediction error without any noising, producing a VIMP value close to zero.

To obtain the confidence interval for VIMP, we used the subsampling approach [7]. We drew a sample, without replacement, with a smaller sample size, from the original data. This sample was used to grow a new forest and calculate VIMP for the covariate. Because we used a small sample, growing a new forest was computationally fast. We repeated this process B times (where B is some large number, B = 100) to obtain estimates of VIMP values. These estimates were used to obtain the standard error for VIMP. Using this standard error and the normality assumption, confidence intervals for VIMP were obtained. These apply similarly to a classical regression model such that a 95% confidence interval (95%C.I.) that does not include 0 contributes meaningfully to the prediction of a response.

One of the main objectives of the RF analysis is to be able to visualize, without model assumptions, the relationship of continuous variables to the response variable. For this, we use the forest to predict the risk-adjusted relationship of selected variable(s) to the response variable. The result is what is known as “partial dependency plots.” Partial plot describes the relationship between the covariate of interest and the response by integrating out (or averaging out or risk-adjusted) the effect of all the other covariates [8]. This thereby provides information about the independent relationship of one covariate on the response assuming all other covariates are constant.

**Model Results**

The outcomes determined from the model are presented in the results section of manuscript. We determined that only surfactant protein B cross the threshold of contributing meaningfully to the prediction of CDH. The results with confidence intervals are detailed in **Table S5**. Additional components of our analysis resulted in a model with a performance error of 0.22 (out-of-bag performance error) and receiver operator characteristic (ROC) curve with an AUC of 0.88.

**Logistic Regression**

Logistic regression is a well-established approach to predicting and classifying observations into binary groups. Model assumptions for logistic regression include linearity of independent variables and log odds and little or no multicollinearity among the independent variables. Although we have shown through the results of our random forest these assumptions are not true for this cohort, logistic regression is much more familiar and straight-forward than our random forest approach. Here we also present the results of a logistic regression model in predicting CDH within this cohort. A logistic regression approach was implemented using the *glm* R-Package [9].

From this model we were able produce a logistic regression model which demonstrated, again, that only surfactant protein B contributed meaningfully to the prediction of CDH. The results with confidence intervals are detailed in **Table S6**. The AIC for this model was 21.0.

We were subsequently then able to determine a ROC curve for our logistic regression (**Figure S6**) which demonstrated a very high AUC of 0.97. This methodology reflects a useful model but does not tell us about what is contributing to the prediction and assumes that the relationship between the covariates and the outcome are linear which we know is not true based on our random forest.

From this ROC curve and data we can create a confusion matrix to determine the specificity and sensitivity of our logistic regression model. This model results in an accuracy of 0.90 (95% CI 0.76 - 0.97), and a 10% misclassification rate. The sensitivity of the model was determined to be 0.91 and specificity of 0.89.

In comparing the random forest to the logistic regression, we see that the logistic regression model makes incorrect assumptions about the relationship of the covariates and overestimates the ability of the ELISA assays to predict CDH.

**Table S1.** Summary information about the samples used in proteomic analysis

|  | **Control early** | **Control term** | **CDH early** | **CDH term** |
| --- | --- | --- | --- | --- |
| n | 12 | 6 | 8 | 14 |
| GA median (SD) | 17 (2,01) | 39 (0,74) | 19,2 (0,85) | 39 (0.74033) |
| Patch |  |  |  | 66,60% |
| ECMO |  |  |  | 13,30% |
| PPHN |  |  |  | 13,30% |
| Survival |  |  |  | 80% |

**Table S2.** Proteins with higher abundance in CDH amniotic fluid.

| **Accession** | **Description** | **Fold change** | **-LOG(P-value)** |
| --- | --- | --- | --- |
| Q16543 | Hsp90 co-chaperone Cdc37 | -4.30276 | 6.587748 |
| P08473 | Neprilysin | -4.17571 | 3.339949 |
| P02792 | Ferritin light chain | -3.41512 | 6.500209 |
| P31997 | Carcinoembryonic antigen-related cell adhesion molecule 8 | -2.97835 | 5.417394 |
| Q96D15 | Reticulocalbin-3 | -2.88371 | 6.629249 |
| Q13753 | Laminin subunit gamma-2 | -2.71466 | 4.320902 |
| Q6UWV6 | Ectonucleotide pyrophosphatase/phosphodiesterase family member 7 | -2.64833 | 3.751885 |
| O95841 | Angiopoietin-related protein 1 | -2.37122 | 3.446454 |
| P10451-3 | Isoform C of Osteopontin | -2.33543 | 2.163047 |
| P24043 | Laminin subunit alpha-2 | -2.31165 | 7.630595 |
| O95467 | Neuroendocrine secretory protein 55 | -2.22372 | 2.721509 |
| Q66K79 | Carboxypeptidase Z | -2.22371 | 2.029689 |
| Q9UHL4 | Dipeptidyl peptidase 2 | -2.2143 | 5.246346 |
| P13611 | Versican core protein | -2.20778 | 4.247535 |
| O75339 | Cartilage intermediate layer protein 1 | -2.18344 | 4.941219 |
| P00488 | Coagulation factor XIII A chain | -2.1575 | 2.557777 |
| Q8IUX7 | Adipocyte enhancer-binding protein 1 | -2.07291 | 4.629464 |
| O75923-13 | Isoform 13 of Dysferlin | -2.00601 | 4.980342 |
| P04179 | Superoxide dismutase [Mn], mitochondrial | -1.95762 | 2.832794 |
| Q9UQ74 | Pregnancy-specific beta-1-glycoprotein 8 | -1.93918 | 2.900568 |
| O14786 | Neuropilin-1 | -1.9234 | 3.11924 |
| P49913 | Cathelicidin antimicrobial peptide | -1.90817 | 2.649524 |
| Q8IWY4 | Signal peptide, CUB and EGF-like domain-containing protein 1 | -1.86942 | 3.479133 |
| O60279 | Sushi domain-containing protein 5 | -1.80072 | 3.52391 |
| P20849 | Collagen alpha-1(IX) chain | -1.80049 | 3.265362 |
| P0DJI8 | Serum amyloid A-1 protein | -1.78069 | 3.184292 |
| P19440 | Glutathione hydrolase 1 proenzyme | -1.74118 | 4.087781 |
| P08582 | Melanotransferrin | -1.74018 | 3.835221 |
| P62328 | Thymosin beta-4 | -1.72398 | 1.624417 |
| Q02388 | Collagen alpha-1(VII) chain | -1.71487 | 4.200001 |
| O14672 | Disintegrin and metalloproteinase domain-containing protein 10 | -1.58066 | 4.668059 |
| P30530 | Tyrosine-protein kinase receptor UFO | -1.50743 | 2.107646 |
| P10915 | Hyaluronan and proteoglycan link protein 1 | -1.50419 | 4.7149 |
| Q13316 | Dentin matrix acidic phosphoprotein 1 | -1.49078 | 4.216441 |
| P10645 | Chromogranin-A | -1.47867 | 3.830841 |
| Q92817 | Envoplakin | -1.47464 | 1.977365 |
| P05997 | Collagen alpha-2(V) chain | -1.45355 | 3.722541 |
| Q5QNW6-2 | Isoform 2 of Histone H2B type 2-F | -1.44942 | 3.411329 |
| P15085 | Carboxypeptidase A1 | -1.43218 | 4.112874 |
| P21333 | Filamin-A | -1.43104 | 5.686266 |
| P21589 | 5'-nucleotidase | -1.39155 | 3.57553 |
| Q9P2E9 | Ribosome-binding protein 1 | -1.38528 | 5.082646 |
| Q16787-1 | Isoform 1 of Laminin subunit alpha-3 | -1.38158 | 3.303071 |
| Q9HBR0 | Putative sodium-coupled neutral amino acid transporter 10 | -1.35724 | 3.089122 |
| Q4ZHG4 | Fibronectin type III domain-containing protein 1 | -1.31545 | 2.889346 |
| P09455-3 | Isoform 3 of Retinol-binding protein 1 | -1.3129 | 2.637172 |
| Q9HD89 | Resistin | -1.30616 | 1.472747 |
| Q04609 | Glutamate carboxypeptidase 2 | -1.26491 | 4.200942 |
| O95969 | Secretoglobin family 1D member 2 | -1.24577 | 3.428108 |
| P10253 | Lysosomal alpha-glucosidase | -1.24055 | 2.129119 |
| Q06828 | Fibromodulin | -1.23991 | 2.595158 |
| P20700 | Lamin-B1 | -1.22569 | 4.7042 |
| P11047 | Laminin subunit gamma-1 | -1.20074 | 3.077985 |
| O00299 | Chloride intracellular channel protein 1 | -1.19408 | 1.93 |
| Q8N6C5-4 | Isoform 4 of Immunoglobulin superfamily member 1 | -1.18865 | 2.854719 |
| P07585 | Decorin | -1.188 | 1.915304 |
| P05387 | 60S acidic ribosomal protein P2 | -1.17733 | 3.306848 |
| P15291 | Beta-1,4-galactosyltransferase 1 | -1.16471 | 2.191514 |
| P84157-2 | Isoform 2 of Matrix-remodeling-associated protein 7 | -1.15604 | 2.322175 |
| Q9Y490 | Talin-1 | -1.15194 | 1.84954 |
| P37173-2 | Isoform 2 of TGF-beta receptor type-2 | -1.10942 | 1.914252 |
| P07307 | Asialoglycoprotein receptor 2 | -1.09399 | 2.069592 |
| P55058 | Phospholipid transfer protein | -1.09026 | 2.627071 |
| Q6UVK1 | Chondroitin sulfate proteoglycan 4 | -1.08124 | 2.986016 |
| Q9UJJ9 | N-acetylglucosamine-1-phosphotransferase subunit gamma | -1.04777 | 1.763076 |
| P28300 | Protein-lysine 6-oxidase | -1.03686 | 1.546975 |
| P07942 | Laminin subunit beta-1 | -1.03608 | 3.635808 |
| Q9H2X0 | Chordin | -1.03485 | 3.151722 |
| P55268 | Laminin subunit beta-2 | -1.03466 | 1.955389 |
| O94985 | Calsyntenin-1 | -1.02858 | 3.355252 |
| A1L4H1 | Soluble scavenger receptor cysteine-rich domain-containing protein SSC5D | -1.0263 | 4.451998 |
| O94907 | Dickkopf-related protein 1 | -1.02471 | 2.087015 |
| Q7L4E1-2 | Isoform 2 of Mitoguardin 2 | -1.01038 | 2.353767 |
| Q9BUD6 | Spondin-2 | -1.00875 | 2.783741 |
| P55291 | Cadherin-15 | -0.99844 | 2.965805 |
| P81172 | Hepcidin | -0.99605 | 3.01139 |
| P09228 | Cystatin-SA | -0.99369 | 1.624363 |
| Q13813-2 | Isoform 2 of Spectrin alpha chain, non-erythrocytic 1 | -0.99131 | 4.047138 |
| Q9UQ72 | Pregnancy-specific beta-1-glycoprotein 11 | -0.9868 | 2.115231 |
| P80370 | Protein delta homolog 1 | -0.97068 | 1.745506 |
| Q9BYF1 | Angiotensin-converting enzyme 2 | -0.96695 | 3.593494 |
| P02655 | Apolipoprotein C-II | -0.96561 | 2.450981 |
| Q9UBH0 | Interleukin-36 receptor antagonist protein | -0.94814 | 1.79288 |
| Q14766 | Latent-transforming growth factor beta-binding protein 1 | -0.90989 | 2.842306 |
| P24821-4 | Isoform 4 of Tenascin | -0.87769 | 3.634541 |
| P21980 | Protein-glutamine gamma-glutamyltransferase 2 | -0.85624 | 1.783238 |
| Q16270 | Insulin-like growth factor-binding protein 7 | -0.83862 | 1.997422 |
| Q13308-6 | Isoform 6 of Inactive tyrosine-protein kinase 7 | -0.82211 | 2.184151 |
| O43278 | Kunitz-type protease inhibitor 1 | -0.81609 | 6.141859 |
| Q07507 | Dermatopontin | -0.80089 | 2.385144 |
| Q12841 | Follistatin-related protein 1 | -0.7442 | 2.165154 |
| P36222 | Chitinase-3-like protein 1 | -0.73979 | 2.212585 |
| Q9UNW1 | Multiple inositol polyphosphate phosphatase 1 | -0.73353 | 2.677265 |
| P13598 | Intercellular adhesion molecule 2 | -0.73346 | 2.578257 |
| Q01638 | Interleukin-1 receptor-like 1 | -0.73207 | 2.044026 |
| Q92520 | Protein FAM3C | -0.73003 | 1.975554 |
| P18206 | Vinculin | -0.71439 | 3.533197 |
| Q86VB7-2 | Isoform 2 of Scavenger receptor cysteine-rich type 1 protein M130 | -0.71239 | 1.903406 |
| P01344-3 | Isoform 3 of Insulin-like growth factor II | -0.70571 | 2.777249 |
| Q9NUQ9 | Protein FAM49B | -0.7001 | 1.846295 |
| Q92692 | Nectin-2 | -0.6951 | 1.827523 |
| P14410 | Sucrase-isomaltase, intestinal | -0.65897 | 3.456989 |
| Q9HCB6 | Spondin-1 | -0.63689 | 1.94646 |
| P22105 | Tenascin-X | -0.6189 | 2.551542 |
| Q02809-2 | Isoform 2 of Procollagen-lysine,2-oxoglutarate 5-dioxygenase 1 | -0.59618 | 2.218829 |
| Q6YHK3 | CD109 antigen | -0.59377 | 2.486932 |
| Q9UMX5 | Neudesin | -0.58036 | 2.446659 |
| P08253 | 72 kDa type IV collagenase | -0.52226 | 2.768078 |
| Q9Y4L1 | Hypoxia up-regulated protein 1 | -0.51038 | 2.203535 |
| Q00888 | Pregnancy-specific beta-1-glycoprotein 4 | -0.5096 | 2.381037 |

**Table S3.** Proteins with higher abundance in control amniotic fluid.

| **Accession** | **Description** | **Difference** | **-LOG(P-value)** |
| --- | --- | --- | --- |
| P02549 | Spectrin alpha chain, erythrocytic 1 | 6.117266 | 4.011321 |
| P17931 | Galectin-3 | 4.172375 | 6.190526 |
| Q969E1 | Liver-expressed antimicrobial peptide 2 | 3.639732 | 5.243736 |
| P00491 | Purine nucleoside phosphorylase | 3.15086 | 5.643956 |
| P00918 | Carbonic anhydrase 2 | 2.970562 | 5.319577 |
| Q9BTY2 | Plasma alpha-L-fucosidase | 2.669027 | 3.164101 |
| O75635 | Serpin B7 | 2.634435 | 3.041501 |
| P68363 | Tubulin alpha-1B chain | 2.533893 | 5.838287 |
| P02788 | Lactotransferrin | 2.461599 | 4.84793 |
| Q9Y6H5-3 | Isoform 3 of Synphilin-1 | 2.441145 | 1.918412 |
| Q6GTX8 | Leukocyte-associated immunoglobulin-like receptor 1 | 2.440083 | 3.222631 |
| P23280 | Carbonic anhydrase 6 | 2.408511 | 4.397925 |
| Q8NFJ5 | Retinoic acid-induced protein 3 | 2.39437 | 2.974245 |
| Q9GZZ8 | Extracellular glycoprotein lacritin | 2.366962 | 4.736435 |
| O00560 | Syntenin-1 | 2.318151 | 3.969116 |
| Q9NS71 | Gastrokine-1 | 2.258946 | 3.069539 |
| O60888-2 | Isoform A of Protein CutA | 2.255175 | 6.638262 |
| Q9NP55 | BPI fold-containing family A member 1 | 2.139128 | 4.649312 |
| Q9UHF0 | Tachykinin-3 | 2.016191 | 4.574237 |
| O43653 | Prostate stem cell antigen | 1.965575 | 2.242232 |
| P60033 | CD81 antigen | 1.872559 | 3.1 |
| P07451 | Carbonic anhydrase 3 | 1.799852 | 4.078605 |
| P31151 | Protein S100-A7 | 1.778672 | 2.908089 |
| P07098-3 | Isoform 3 of Gastric triacylglycerol lipase | 1.736712 | 2.87661 |
| P02679 | Fibrinogen gamma chain | 1.732303 | 2.027447 |
| P25940 | Collagen alpha-3(V) chain | 1.721801 | 1.394006 |
| P13489 | Ribonuclease inhibitor | 1.697689 | 3.069441 |
| Q86UD1 | Out at first protein homolog | 1.581485 | 2.210841 |
| P29401-2 | Isoform 2 of Transketolase | 1.575579 | 1.587057 |
| P22891-2 | Isoform 2 of Vitamin K-dependent protein Z | 1.569987 | 2.974372 |
| P00709 | Alpha-lactalbumin | 1.564791 | 1.601135 |
| O43895 | Xaa-Pro aminopeptidase 2 | 1.558845 | 3.441821 |
| Q8NFL0 | UDP-GlcNAc:betaGal beta-1,3-N-acetylglucosaminyltransferase 7 | 1.544606 | 2.961442 |
| O94910 | Adhesion G protein-coupled receptor L1 | 1.527593 | 3.632196 |
| P05089-2 | Isoform 2 of Arginase-1 | 1.508801 | 4.260408 |
| P59665 | Neutrophil defensin 1 | 1.507616 | 2.623608 |
| P19801-2 | Isoform 2 of Amiloride-sensitive amine oxidase [copper-containing] | 1.495286 | 1.923867 |
| P15586 | N-acetylglucosamine-6-sulfatase | 1.460791 | 5.210591 |
| P68104 | Elongation factor 1-alpha 1 | 1.448132 | 1.611766 |
| P31150 | Rab GDP dissociation inhibitor alpha | 1.422066 | 3.542265 |
| P04114 | Apolipoprotein B-100 | 1.416851 | 2.777067 |
| P02808 | Statherin | 1.409262 | 3.75018 |
| P13473-3 | Isoform LAMP-2C of Lysosome-associated membrane glycoprotein 2 | 1.400626 | 2.329481 |
| P60903 | Protein S100-A10 | 1.399215 | 1.51344 |
| Q9Y6C2 | EMILIN-1 | 1.386192 | 4.103133 |
| Q9UGT4 | Sushi domain-containing protein 2 | 1.385951 | 3.471872 |
| P31025 | Lipocalin-1 | 1.370589 | 1.893252 |
| P04745 | Alpha-amylase 1 | 1.326274 | 1.929251 |
| P01023 | Alpha-2-macroglobulin | 1.318868 | 4.627027 |
| P04155 | Trefoil factor 1 | 1.315485 | 1.724821 |
| Q13510-2 | Isoform 2 of Acid ceramidase | 1.312937 | 2.080418 |
| Q8IUL8 | Cartilage intermediate layer protein 2 | 1.304286 | 3.560172 |
| P05164-3 | Isoform H7 of Myeloperoxidase | 1.294223 | 3.218739 |
| P05154 | Plasma serine protease inhibitor | 1.243631 | 4.504379 |
| P62805 | Histone H4 | 1.221811 | 1.982757 |
| Q8IW75 | Serpin A12 | 1.221464 | 3.733802 |
| P07988 | Pulmonary surfactant-associated protein B | 1.22 | 1.884052 |
| Q8WXI7 | Mucin-16 | 1.199303 | 1.530876 |
| Q15782 | Chitinase-3-like protein 2 | 1.195141 | 2.178684 |
| P15941-2 | Isoform 2 of Mucin-1 | 1.193224 | 3.213912 |
| P54108-3 | Isoform 3 of Cysteine-rich secretory protein 3 | 1.166524 | 2.002456 |
| P19652 | Alpha-1-acid glycoprotein 2 | 1.159539 | 1.859639 |
| Q8WVQ1 | Soluble calcium-activated nucleotidase 1 | 1.15927 | 3.615697 |
| P0DME0 | Protein SETSIP | 1.134629 | 2.43666 |
| Q6E0U4-2 | Isoform 2 of Dermokine | 1.129937 | 2.932571 |
| P16104 | Histone H2AX | 1.127474 | 2.477432 |
| P14618 | Pyruvate kinase PKM | 1.126662 | 2.135333 |
| P30043 | Flavin reductase (NADPH) | 1.123076 | 3.00941 |
| P35237 | Serpin B6 | 1.10905 | 3.207397 |
| P29508 | Serpin B3 | 1.100711 | 1.85047 |
| P22792 | Carboxypeptidase N subunit 2 | 1.100219 | 2.604397 |
| P04003 | C4b-binding protein alpha chain | 1.046104 | 4.393267 |
| Q9BPY8-3 | Isoform 3 of Homeodomain-only protein | 1.03652 | 2.076622 |
| P20142 | Gastricsin | 1.033158 | 1.949625 |
| P35625 | Metalloproteinase inhibitor 3 | 1.030421 | 2.645072 |
| P05362 | Intercellular adhesion molecule 1 | 1.025355 | 2.086781 |
| P80303 | Nucleobindin-2 | 1.022768 | 1.850204 |
| Q9HC38-2 | Isoform 2 of Glyoxalase domain-containing protein 4 | 1.016191 | 1.850321 |
| P07498 | Kappa-casein | 1.007618 | 1.96567 |
| P00558 | Phosphoglycerate kinase 1 | 0.995792 | 2.433785 |
| Q15109-4 | Isoform 4 of Advanced glycosylation end product-specific receptor | 0.994694 | 1.759203 |
| P07108-5 | Isoform 5 of Acyl-CoA-binding protein | 0.991811 | 2.1195 |
| P05186 | Alkaline phosphatase, tissue-nonspecific isozyme | 0.983214 | 3.702681 |
| P52823 | Stanniocalcin-1 | 0.947788 | 1.849062 |
| P04792 | Heat shock protein beta-1 | 0.941978 | 1.859355 |
| P05121 | Plasminogen activator inhibitor 1 | 0.941035 | 1.595999 |
| Q13228-4 | Isoform 4 of Methanethiol oxidase | 0.904836 | 2.336376 |
| Q06033 | Inter-alpha-trypsin inhibitor heavy chain H3 | 0.891863 | 1.914731 |
| P14780 | Matrix metalloproteinase-9 | 0.885801 | 1.714418 |
| O43505 | Beta-1,4-glucuronyltransferase 1 | 0.856096 | 1.772535 |
| Q96QR1 | Secretoglobin family 3A member 1 | 0.853035 | 2.420033 |
| O60259-2 | Isoform 2 of Kallikrein-8 | 0.825592 | 2.159681 |
| Q9BU40-4 | Isoform 4 of Chordin-like protein 1 | 0.824088 | 1.890127 |
| P27348 | 14-3-3 protein theta | 0.811349 | 1.727707 |
| P15328 | Folate receptor alpha | 0.803851 | 1.773688 |
| O60462 | Neuropilin-2 | 0.803719 | 2.501717 |
| P53634 | Dipeptidyl peptidase 1 | 0.786234 | 2.026308 |
| P05090 | Apolipoprotein D | 0.777767 | 2.686589 |
| Q9UIV8 | Serpin B13 | 0.751068 | 1.891646 |
| Q9H299 | SH3 domain-binding glutamic acid-rich-like protein 3 | 0.745396 | 2.341073 |
| P07195 | L-lactate dehydrogenase B chain | 0.731666 | 2.088086 |
| O00391 | Sulfhydryl oxidase 1 | 0.72486 | 2.046141 |
| O00339 | Matrilin-2 | 0.694823 | 2.170377 |
| P13639 | Elongation factor 2 | 0.69479 | 1.84969 |
| P14384 | Carboxypeptidase M | 0.682113 | 2.063786 |
| Q96CG8 | Collagen triple helix repeat-containing protein 1 | 0.665641 | 2.228228 |
| O75144-2 | Isoform 2 of ICOS ligand | 0.641118 | 1.932461 |
| P02751-8 | Isoform 8 of Fibronectin | 0.560048 | 2.061528 |

**Table S4.** Proteins identified in only one cohort but in all samples within that cohort.

| **Accession No.** | **Protein ID** | **Fold Change**  **CDH/Control** |
| --- | --- | --- |
| O94886 | CSC1-like protein 1 | -6.64 |
| Q8TD57 | Dynein heavy chain 3, axonemal | -6.64 |
| P08581-2 | Isoform 2 of Hepatocyte growth factor receptor | -6.64 |
| P36269-3 | Isoform 3 of Glutathione hydrolase 5 proenzyme | -6.64 |
| Q9Y337 | Kallikrein-5 | -6.64 |
| O43451 | Maltase-glucoamylase, intestinal | -6.64 |
| Q02325 | Plasminogen-like protein B | -6.64 |
| Q15149 | Plectin | -6.64 |
| P01133 | Pro-epidermal growth factor | -6.64 |
| P20396 | Pro-thyrotropin-releasing hormone | -6.64 |
| P11717 | Cation-independent mannose-6-phosphate receptor | 6.64 |
| Q9H9P2 | Chondrolectin | 6.64 |
| Q12860 | Contactin-1 | 6.64 |
| P27487 | Dipeptidyl peptidase 4 | 6.64 |
| Q29960 | HLA class I histocompatibility antigen, Cw-16 alpha chain | 6.64 |
| P43626-2 | Isoform 2 of Killer cell immunoglobulin-like receptor 2DL1 | 6.64 |
| P05556-3 | Isoform 3 of Integrin beta-1 | 6.64 |
| P43628 | Killer cell immunoglobulin-like receptor 2DL3 | 6.64 |
| Q6UX15 | Layilin | 6.64 |
| Q9NR99 | Matrix-remodeling-associated protein 5 | 6.64 |
| P13640 | Metallothionein-1G | 6.64 |
| P16860 | Natriuretic peptides B | 6.64 |
| P48594 | Serpin B4 | 6.64 |
| P61278 | Somatostatin | 6.64 |
| P07951 | Tropomyosin beta chain | 6.64 |

**Table S5**. Random Forest point estimate and confidence intervals of variable of important. A subsampling approach without replacement was used to approximate 95% confidence intervals for each variable within the model.

| **Variable** | **Point Estimate** | **95% Confidence Interval** |
| --- | --- | --- |
| Surfactant Protein B | 16 | (2.7 – 29) |
| Osteopontin | 2.4 | (-0.8 – 5.5) |
| Kallikrein 5 | 3.5 | (-2.3 – 9.3) |
| Galectin-3 | 2.3 | (-3.0 – 7.6) |
| Time of Amniotic Fluid Collection | -0.7 | (-6.7 – 5.4) |

**Table S6**. Logistic regression point estimate and confidence intervals of variable of importance.

| **Variable** | **Point Estimate** | **95% Confidence Interval** | **P-Value** |
| --- | --- | --- | --- |
| Intercept | 2.57 | (1.3 – 2.4) | 0.12 |
| Surfactant Protein B | 0.64 | (0.33 – 0.86) | 0.04 |
| Osteopontin | 1.4 | (1.1– 2.8) | 0.10 |
| Kallikrein 5 | 4.5x10^-7^ | (0.00 - 0.12) | 0.57 |
| Galectin-3 | 1.3 | (0.58 – 4.6) | 0.60 |
| Time of Amniotic Fluid Collection (Term) | 5.9 | (0.57 – 9.3) | 0.57 |


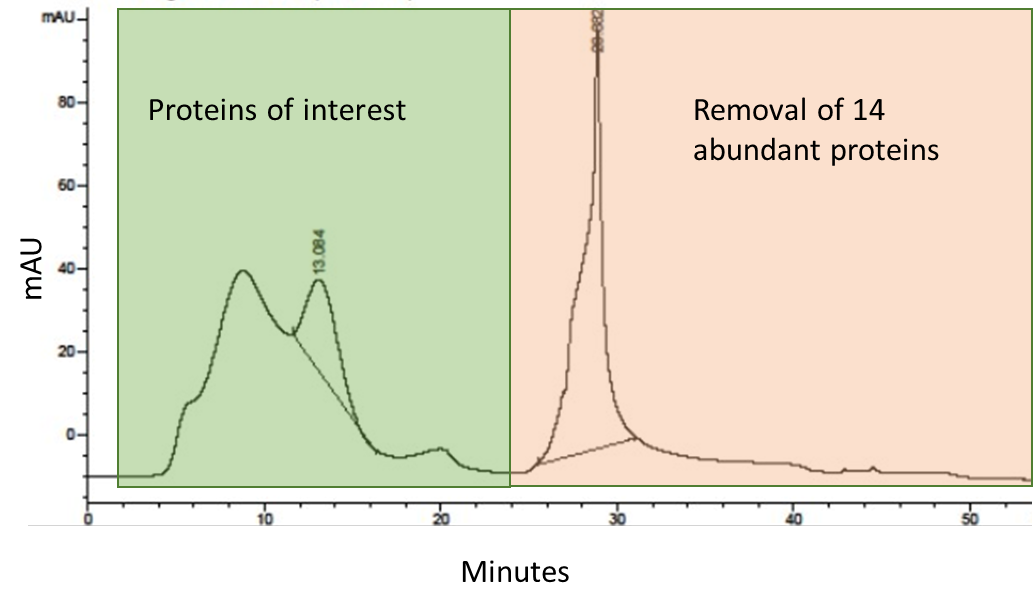


**Figure S1:**  Time-resolved chromatogram of amniotic fluid proteins subjected to depletion by a Seppro-Supermix LC5 column. The green-shaded region illustrates the (non-binding) fractions eluting from 5-25 mins, which were retained and used for label-free proteomics analysis whereas the region shaded in pink shows the elution peak of 14 abundant proteins that bound to the column, which were subsequently discarded. This reduced the sample dynamic range and increased the likelihood of detecting low abundance proteins.


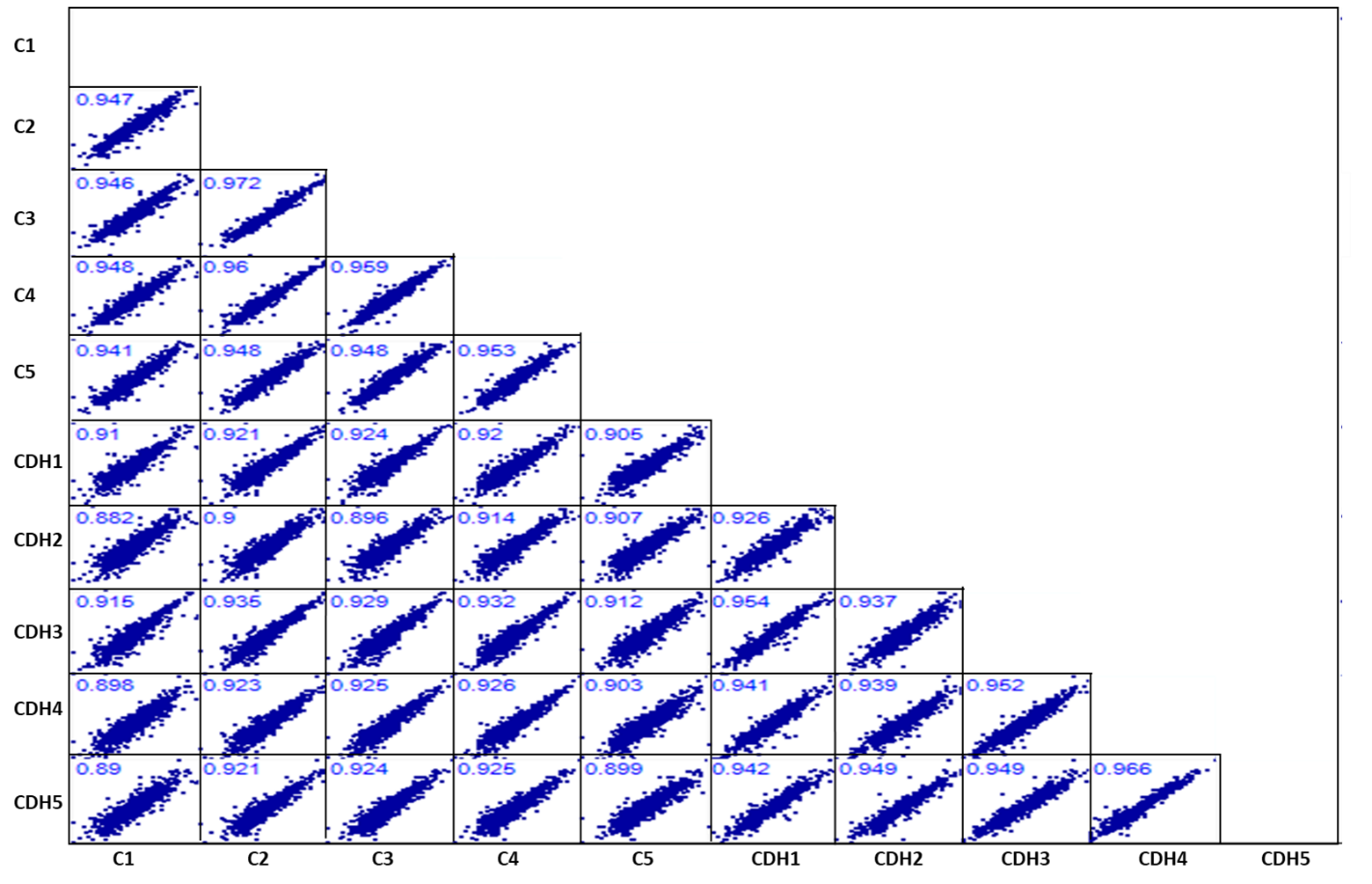


# Figure S2: Statistical analysis of all the identified proteins using Perseus software. A multi-scatter plot was generated to determine correlation of the proteins within and between the control and CDH amniotic fluid cohorts collected at term. Pearson correlation coefficient values are shown for each sample in the block.

#
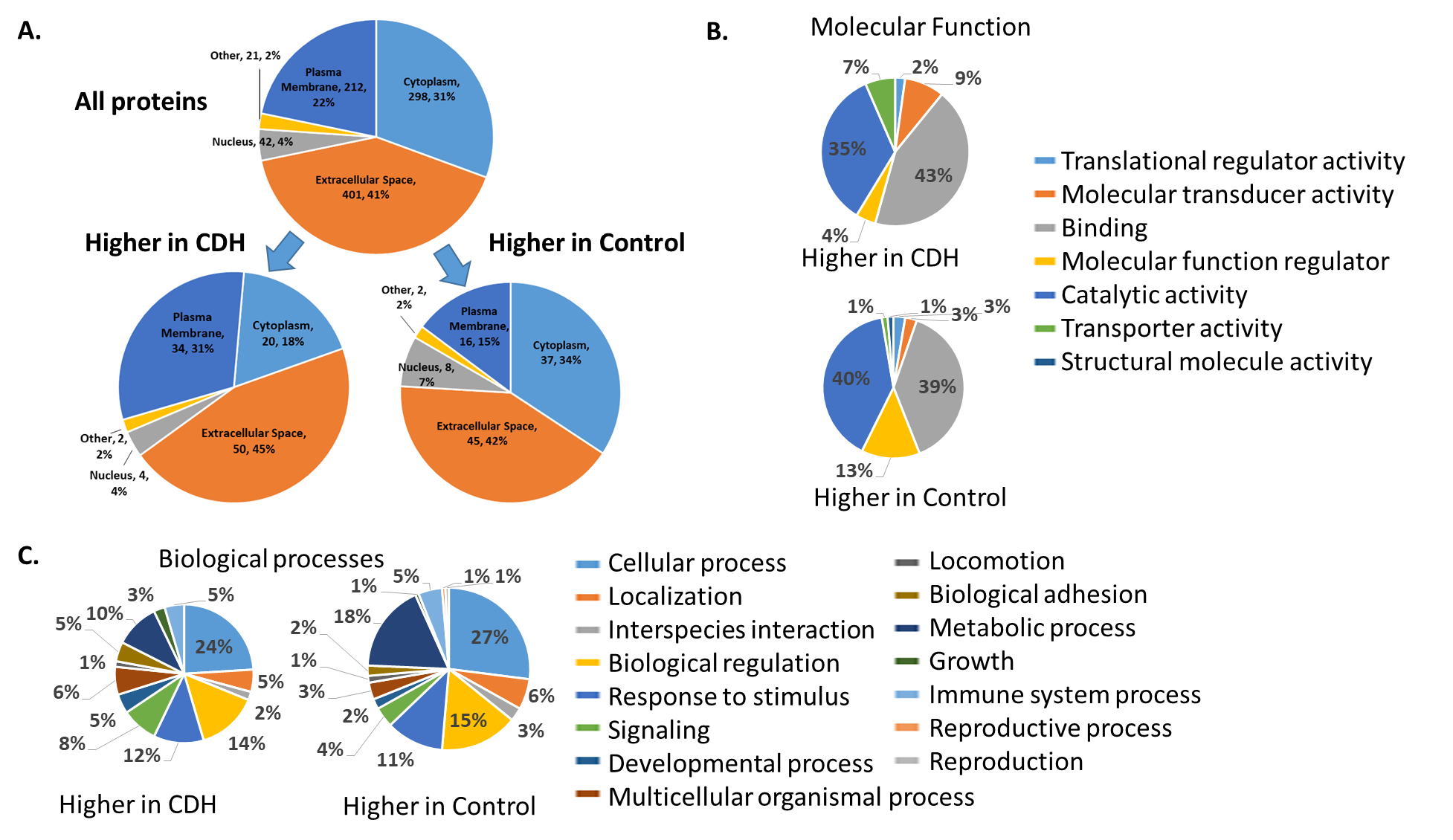


**Figure S3:** Pie chart assigning the differentially abundant amniotic fluid proteins to groups according to **A.** Cellular components, **B**. Molecular function and **C.** Biological processes.


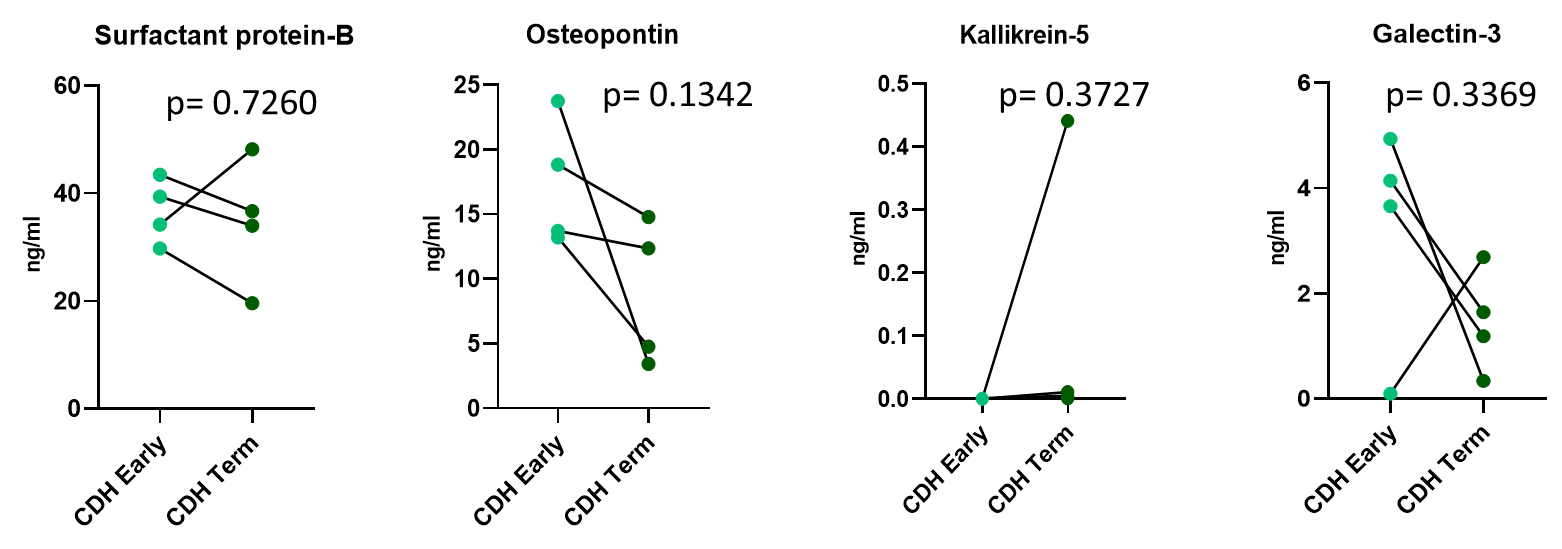


**Figure S4:** Dumbbell plots show the levels of select proteins in amniotic fluid collected from women carrying fetuses with CDH collected during both the second trimester and at term. A paired t-test was applied to determine statistical significance.


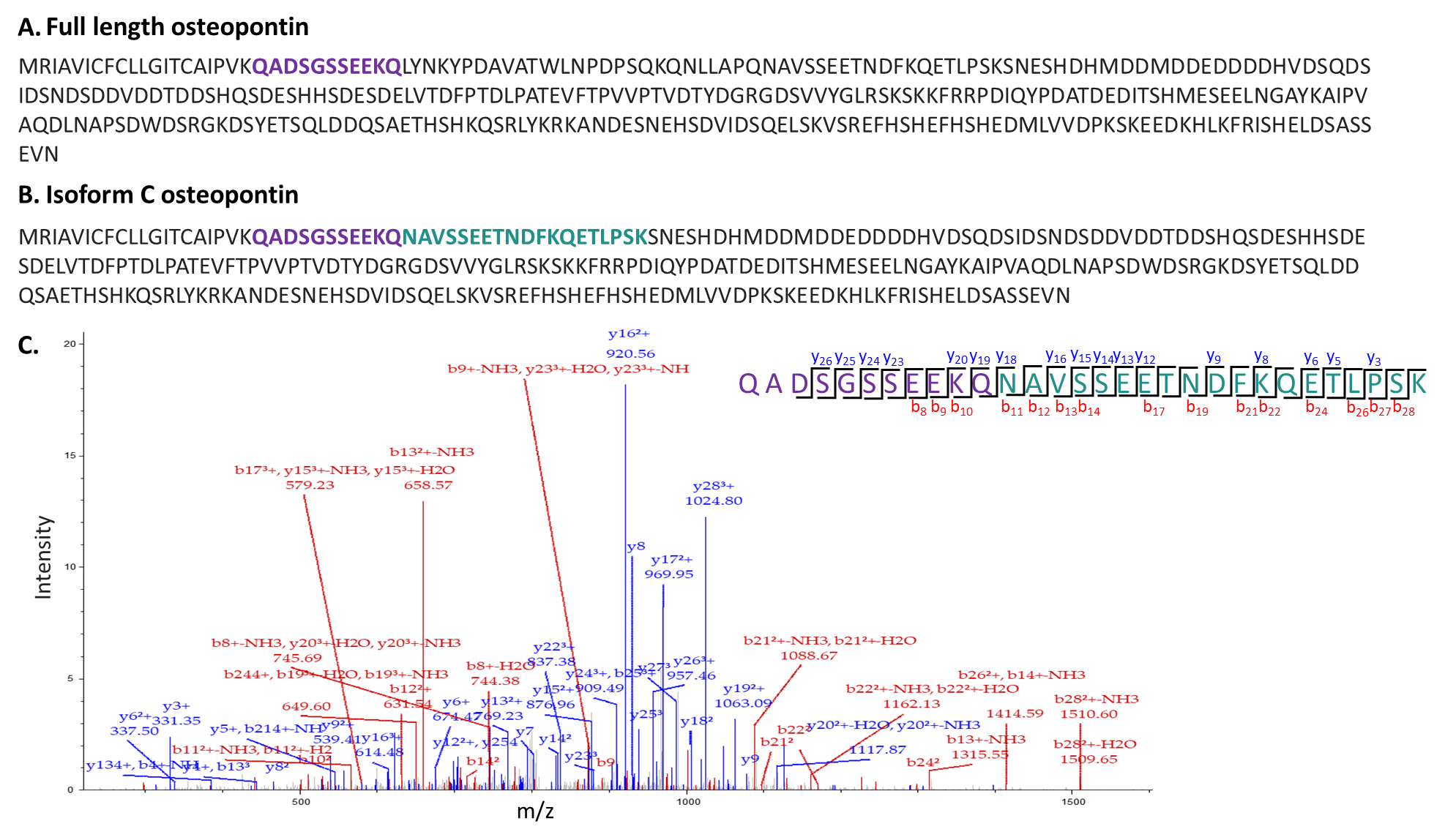


**Figure S5: A.** Canonical sequence of osteopontin (isoform a), **B.** Sequence of osteopontin isoform c and **C.** MS^2^ spectrum of a quadruply charged osteopontin isoform c-specific peptide with two missed cleavages and Sequest Xcorr score of 3.39. Purple and green text correspond to osteopontin sequences similarly marked in A and B.


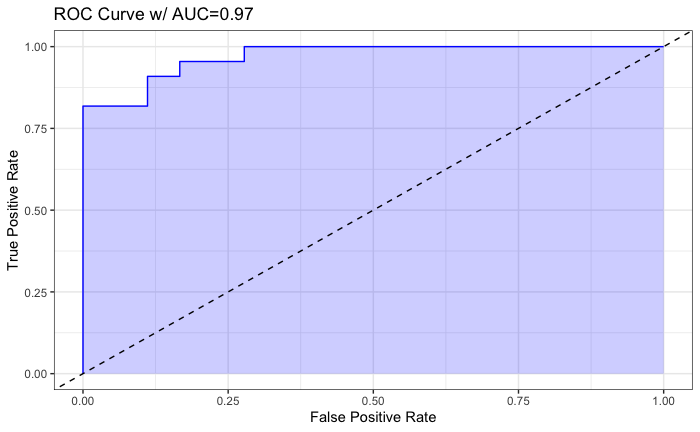


**Figure S6**. Receiver operator characteristic (ROC) curve for logistic regression predicting CDH. *AUC* – Area under the curve.

References:

1. Breiman L: Random forests. Machine Learning 2001;45:5-32.
2. Breiman L, Friedman JH, Olshen RA, Stone CJ. Classification and regression trees. Boca Raton, FL: CRC Press; 1984.
3. R Core Team. R: A language and environment for statistical computing. R: Foundation for Statistical Computing, Vienna, Austria. 2018 (Accessed 2019, at http://www.R-project.org.)
4. Ishwaran H, Kogalur UB. RandomForestSRC: Random forests for survival, regression and classification (RF-SRC). R package version 2.5.0. http://cran.r-project.org, 2017.
5. Ishwaran H. (2007) Variable importance in binary regression trees and forests. Electron J Statist;1:519-37.
6. Ishwaran H. and Lu M. (2019). Standard errors and confidence intervals for variable importance in random forest regression, classification, and survival. Statistics in Medicine, 38, 558-582.
7. Ishwaran H, Lu M. Standard errors and confidence intervals for variable importance in random forest regression, classification, and survival. Stat Med. 2019;38:558-582.
8. Friedman JH. Greedy Function Approximation: A Gradient Boosting Machine. Ann Stat. 2000;29:1189-1232.
9. Dobson AJ, and Barnett AG. An introduction to generalized linear models. Chapman and Hall/CRC; 2018.
