# Supplementary material for: Identification of protein biomarkers associated with congenital diaphragmatic hernia in human amniotic fluid
Source: Sci Rep. 2023 Sep 19;13:15483. doi: 10.1038/s41598-023-42576-2 (PMC10509251; doi:10.1038/s41598-023-42576-2)
Supplement: Supplementary file 3 — Supplementary Information 3. [file 41598_2023_42576_MOESM3_ESM.html]

CDH Biomarker Analysis


Code 

- Show All Code
- Hide All Code

# CDH Biomarker Analysis

## CDH Biomarker Analysis

- 1 Loading the Raw Data into
  R
- 2 Evaluating Data
  - 2.1 Overall
  - 2.2 Visualizing
    Surfactant-B
  - 2.3 Visualizing
    Osteopontin
  - 2.4 Visualizing
    Kallikein-5
  - 2.5 Visualizing
    Galectin-3
  - 2.6 Missing Data
- 3 Table Overall
- 4 Table Early
- 5 Table Term
- 6 Predication Model - Logistic
  Regression
  - 6.1 Visualize Surfactant-B
    Data
- 7 ROC For Logistic
  Regression
- 8 Random Forest
  - 8.1 RF Setting
  - 8.2 Start RF - Overall
  - 8.3 RF OOB Error
  - 8.4 Variable Selection
  - 8.5 VIMP Plots
  - 8.6 VIMP Plots (Labelled
    Figure)
  - 8.7 Dependence Plots
  - 8.8 Partial Plots
  - 8.9 Stratify by Time
  - 8.10 Checking Incorrect
    Assignments
- 9 Conclusion
- 10 Manuscript Figures
  - 10.1 Variable of Important
    (VIMP) Plot
  - 10.2 Surfactant B Partial
    Plot

Benjamin
Kramer

2022-12-19

```
## Global options
library(knitr); library(rmdformats)
  opts_chunk$set(comment=NA)
  opts_knit$set(width=75)
```

```
library(here); library(janitor); library(magrittr)
library(rms); library(broom); library(naniar); 
library(simputation); library(ROCR)
library(caret); library(car); library(GGally);
library(broom); library(rsample);
library(yardstick); library(readxl)
library(rms); library(equatiomatic); library(haven); library(sandwich); 
library(boot); library(countreg); library(lars); library(ggplot2)
library(ggrepel); library(readxl); library(lubridate); library(xlsx);
library(RColorBrewer); library(survminer); library(tidyverse); library(mice);
library(tableone); library(DataExplorer); library(boostmtree);
library(foreign); library(randomForestSRC); library(rJava);
library(BoostMLR); library(RColorBrewer);       # John's plotting package, adjust plots for PPT format etc.
library(reshape2); library(lemon); library(equatiomatic); library(glue);
library(conflicted); library(ggRandomForests)

conflict_prefer("select", "dplyr")
conflict_prefer("filter", "dplyr")


theme_set(theme_bw())
```

# 1 Loading the Raw Data into R

Here we load in the data into R and clean it a bit. We will also
evaluate the type of variables and make sure we have everything coded as
desired.

```
# data
dat_cdh <- read_xlsx("sumit_analysis.xlsx") %>%
#clean variable names
  clean_names() %>% 
#abbrv. names to desired names
  rename(surf_b = surfactant_b_ng_ml, 
          osteop = osteopontin_ng_ml, 
          kalli_5 = kallikrein_5_ng_ml, 
          gale_3 = galectin_3_ng_ml, 
         id = patient_id) %>% 
#convert varialbe types
  type.convert(as.is = FALSE) %>% 
#make appropriate variables factors and set baseline levels
  mutate(id = as.factor(id), 
         disease = fct_relevel(disease, "Control"), 
         time =  fct_relevel(time, "Early"))

#assess data structure
str(dat_cdh)
```

```
tibble [40 � 7] (S3: tbl_df/tbl/data.frame)
 $ id     : Factor w/ 40 levels "15","16","17",..: 3 4 7 21 35 37 2 5 6 9 ...
 $ disease: Factor w/ 2 levels "Control","CDH": 1 1 1 1 1 1 1 1 1 1 ...
 $ time   : Factor w/ 2 levels "Early","Term": 2 2 2 2 2 2 1 1 1 1 ...
 $ surf_b : num [1:40] 42.8 44.1 36.7 29.8 44.4 ...
 $ osteop : num [1:40] 3.09771 6.39818 6.91726 6.90177 0.00643 ...
 $ kalli_5: num [1:40] 3.43 4.554 1.191 3.997 0.798 ...
 $ gale_3 : num [1:40] 4.15 10.11 1.34 5.23 7.06 ...
```

This confirms the data is loaded in correctly

# 2 Evaluating Data

## 2.1 Overall

Data Summary

```
Hmisc::describe(dat_cdh)
```

```
dat_cdh 

 7  Variables      40  Observations
--------------------------------------------------------------------------------
id 
       n  missing distinct 
      40        0       40 

lowest : 15 16 17 18 19, highest: 65 67 68 73 79
--------------------------------------------------------------------------------
disease 
       n  missing distinct 
      40        0        2 
                          
Value      Control     CDH
Frequency       18      22
Proportion    0.45    0.55
--------------------------------------------------------------------------------
time 
       n  missing distinct 
      40        0        2 
                      
Value      Early  Term
Frequency     20    20
Proportion   0.5   0.5
--------------------------------------------------------------------------------
surf_b 
       n  missing distinct     Info     Mean      Gmd      .05      .10 
      40        0       40        1    41.23    10.85    26.53    29.44 
     .25      .50      .75      .90      .95 
   35.11    42.13    48.75    51.67    52.90 

lowest : 19.56862 25.49964 26.58843 28.27892 29.56827
highest: 51.60189 52.28003 52.69068 56.87391 62.51842
--------------------------------------------------------------------------------
osteop 
       n  missing distinct     Info     Mean      Gmd      .05      .10 
      40        0       40        1     11.9    7.983    2.954    3.718 
     .25      .50      .75      .90      .95 
   6.896   11.399   17.378   22.326   22.906 

lowest :  0.006427  0.225440  3.097709  3.438602  3.748505
highest: 22.272950 22.799790 22.861770 23.737240 24.690200
--------------------------------------------------------------------------------
kalli_5 
        n   missing  distinct      Info      Mean       Gmd       .05       .10 
       40         0        32     0.999    0.4743    0.8283 0.0001340 0.0001970 
      .25       .50       .75       .90       .95 
0.0003425 0.0008440 0.2318770 1.3029631 3.4584428 

lowest : 0.000104 0.000134 0.000188 0.000198 0.000263
highest: 1.190868 2.311819 3.430083 3.997279 4.553722
                                                                            
Value       0.00  0.01  0.18  0.23  0.44  0.65  0.80  0.92  1.19  2.31  3.43
Frequency     26     2     1     2     1     1     1     1     1     1     1
Proportion 0.650 0.050 0.025 0.050 0.025 0.025 0.025 0.025 0.025 0.025 0.025
                      
Value       4.00  4.55
Frequency      1     1
Proportion 0.025 0.025

For the frequency table, variable is rounded to the nearest 0.01
--------------------------------------------------------------------------------
gale_3 
       n  missing distinct     Info     Mean      Gmd      .05      .10 
      40        0       40        1    3.922    3.657   0.0911   0.3200 
     .25      .50      .75      .90      .95 
  1.4002   3.3564   6.4663   8.5818   8.9277 

lowest : -0.093980  0.014093  0.095151  0.154593  0.338323
highest:  8.557532  8.800704  8.865550 10.108430 13.026490
--------------------------------------------------------------------------------
```

From this we see that we have 40 disctinct patients. 22 (55%) with
from mother with children wtih CDH and 18 (45%) from control mothers
(children without CDH). There are 20 patients in each group 20 (50%)
early samples and 20 (50%) term samples. Per the details provided these
are observations from 40 distinct patients. Below is the utilized data
dictionary.

| Variable | Type | Description / Levels |
| --- | --- | --- |
| `id` | Patient ID | subject code (15-79) |
| `disease` | Factor (2-Levels) | CDH, Control: Based on whether the mother had a child with CDH or without CDH (CDH = Congenital Diaphragmatic Hernia) |
| `time` | Factor (2-Levels) | Early, Term: Time point at which the blood was taken |
| `surf_b` | Quant | Level in ng/dl of surfactant-b in ELISA Assay. Cannot be a negative number |
| `osteopontin` | Quant | Level in ng/dl of osteopontin in ELISA Assay. Cannot be a negative number |
| `kali_5` | Quant | Level in ng/dl of kllikrein-5 in ELISA Assay. Cannot be a negative number |
| `gale_3` | Quant | Level in ng/dl of Galectin-3 in ELISA Assay. Cannot be a negative number |

How lets evaluate our quantitative variables for missing data or
errors

## 2.2 Visualizing Surfactant-B

```
#Histogram of data
dat_cdh %>% 
ggplot(aes(x = surf_b)) +
    geom_histogram(fill = "slateblue", col = "white", 
                   binwidth = 2) + 
    labs(x = "Surfactant-B Concentration (ng/dL)") +
    theme_bw()
```

```
#Normal QQ Plot
dat_cdh %>% 
ggplot(aes(sample = surf_b)) +
    geom_qq(col = "slateblue") + geom_qq_line(col = "red") + 
    labs(y = "Surfactant-B Concentration (ng/dL)") +
    theme_bw()
```

```
dat_cdh %>% 
ggplot(aes(x = disease,y = surf_b)) +
  geom_boxplot(width = 0.5) +
  geom_jitter(aes(col = time), width = 0.1) +
  guides() +
  theme_classic() +
  labs(y = "Sufactant-B Levels (mg/dL)")
```

We see here that all values are positive and the distribution is
relatively normal. N0 editing is required. We also see here that we can
visualize that patients with CDH children have a lower level of
Surfactant B. There is no clear relationship with time based on this
figure.

## 2.3 Visualizing Osteopontin

```
#Histogram of data
dat_cdh %>% 
ggplot(aes(x = osteop)) +
    geom_histogram(fill = "slateblue", col = "white", 
                   binwidth = 2) + 
    labs(x = "Osteopontin Concentration (ng/dL)") +
    theme_bw()
```

```
#Normal QQ Plot
dat_cdh %>% 
ggplot(aes(sample = osteop)) +
    geom_qq(col = "slateblue") + geom_qq_line(col = "red") + 
    labs(y = "Ostepontin Concentration (ng/dL)") +
    theme_bw()
```

```
dat_cdh %>% 
ggplot(aes(x = disease,y = osteop)) +
  geom_boxplot(width = 0.5, outlier.shape = NA) +
  geom_jitter(aes(color = time), width = 0.1) +
  guides() +
  theme_classic() +
  labs(y = "Ostepontin Concentration (ng/dL)")
```

We see here that all values are positive and the distribution is
relatively normal. No editing is required. We also see that Osteopontin
concentration tends to be higher in the mothers of children with CDH. We
also see that these concentrations tend to be higher early (Early)
vs.�later (Term).

## 2.4 Visualizing Kallikein-5

```
#Histogram of data
dat_cdh %>% 
ggplot(aes(x = kalli_5)) +
    geom_histogram(fill = "slateblue", col = "white", 
                   binwidth = 0.2) + 
    labs(x = "Osteopontin Concentration (ng/dL)") +
    theme_bw()
```

```
#Normal QQ Plot
dat_cdh %>% 
ggplot(aes(sample = kalli_5)) +
    geom_qq(col = "slateblue") + geom_qq_line(col = "red") + 
    labs(y = "Kallikrein-5 Concentration (ng/dL)") +
    theme_bw()
```

```
dat_cdh %>% 
ggplot(aes(x = disease,y = kalli_5)) +
  geom_boxplot(outlier.shape = NA, width = 0.5) +
  geom_jitter(aes(col = time) , width = 0.1) +
  guides() +
  theme_classic() +
  labs(y = "Kallikrein-5 Concentration (ng/dL)")
```

We see here that all values are positive. But the distribution is
heavily right skewed by some outliers. No editing is required for this,
but normality assumptions may be limited. For the sake of our analysis
we will not transform this variable.

Mother of children with CDH tended to have lower CDH levels. We can
also see here there is a clear time component with Kallikrein-5. Such
that at early times Kallirein-5 is usually close ot zero.

## 2.5 Visualizing Galectin-3

```
#Histogram of data
dat_cdh %>% 
ggplot(aes(x = gale_3)) +
    geom_histogram(fill = "slateblue", col = "white", 
                   binwidth = 2) + 
    labs(x = "Galectin-3 Concentration (ng/dL)") +
    theme_bw()
```

```
#Normal QQ Plot
dat_cdh %>% 
ggplot(aes(sample = gale_3)) +
    geom_qq(col = "slateblue") + geom_qq_line(col = "red") + 
    labs(y = "Galectin-3 Concentration (ng/dL)") +
    theme_bw()
```

```
dat_cdh %>% 
ggplot(aes(x = disease,y = gale_3)) +
  geom_boxplot(outlier.shape = NA, width = 0.5) +
  geom_jitter(aes(col = time), width = 0.1) +
  guides() +
  theme_classic() +
  labs(y = "Galectin-3 Concentration (ng/dL)")
```

We see here that NOT all values are positive. In discussion with
Sumit, any values < 0 should be interpreted as 0. So that a negative
outcome of the ELISA assay for concentration cannot be less than 0.

```
dat_cdh %>% 
  mutate(gale_3 = if_else(gale_3 < 0, 0, gale_3)) -> dat_cdh

Hmisc::describe((dat_cdh$gale_3))
```

```
(dat_cdh$gale_3) 
       n  missing distinct     Info     Mean      Gmd      .05      .10 
      40        0       40        1    3.924    3.652   0.0911   0.3200 
     .25      .50      .75      .90      .95 
  1.4002   3.3564   6.4663   8.5818   8.9277 

lowest :  0.000000  0.014093  0.095151  0.154593  0.338323
highest:  8.557532  8.800704  8.865550 10.108430 13.026490
```

Now the data has been corrected

## 2.6 Missing Data

```
miss_var_summary(dat_cdh)
```

```
# A tibble: 7 � 3
  variable n_miss pct_miss
  <chr>     <int>    <dbl>
1 id            0        0
2 disease       0        0
3 time          0        0
4 surf_b        0        0
5 osteop        0        0
6 kalli_5       0        0
7 gale_3        0        0
```

There is no missing data in this report and thus, a complete case
analysis is appropriate.

# 3 Table Overall

```
vars <- c("surf_b", "osteop", "kalli_5", "gale_3")
trt <- c("disease")

table_1 <- CreateTableOne(data = dat_cdh,
                          vars = vars,
                          strata = trt,
                          includeNA = FALSE, 
                          test = FALSE)

print(table_1)
```

```
                     Stratified by disease
                      Control      CDH         
  n                      18           22       
  surf_b (mean (SD))  47.24 (7.40) 36.30 (8.05)
  osteop (mean (SD))   9.75 (6.15) 13.67 (7.11)
  kalli_5 (mean (SD))  0.90 (1.56)  0.12 (0.25)
  gale_3 (mean (SD))   5.46 (3.68)  2.67 (2.25)
```

# 4 Table Early

```
dat_cdh %>% filter(time == "Early") -> dat_cdh_early

vars <- c("surf_b", "osteop", "kalli_5", "gale_3")
trt <- c("disease")

table_early <- CreateTableOne(data = dat_cdh_early,
                          vars = vars,
                          strata = trt,
                          includeNA = FALSE, 
                          test = FALSE)

print(table_early)
```

```
                     Stratified by disease
                      Control      CDH         
  n                      12            8       
  surf_b (mean (SD))  50.03 (5.72) 39.16 (6.33)
  osteop (mean (SD))  11.98 (6.13) 18.57 (3.84)
  kalli_5 (mean (SD))  0.00 (0.00)  0.00 (0.00)
  gale_3 (mean (SD))   4.78 (3.37)  3.58 (2.73)
```

Table 1. Showing Concentrations between two groups when stratified by
Disease and time.

# 5 Table Term

```
dat_cdh %>% filter(time == "Term") -> dat_cdh_term

vars <- c("surf_b", "osteop", "kalli_5", "gale_3")
trt <- c("disease")

table_term <- CreateTableOne(data = dat_cdh_term,
                          vars = vars,
                          strata = trt,
                          includeNA = FALSE, 
                          test = FALSE)

print(table_term)
```

```
                     Stratified by disease
                      Control      CDH         
  n                       6           14       
  surf_b (mean (SD))  41.67 (7.65) 34.67 (8.68)
  osteop (mean (SD))   5.27 (3.11) 10.87 (7.10)
  kalli_5 (mean (SD))  2.71 (1.53)  0.19 (0.29)
  gale_3 (mean (SD))   6.82 (4.22)  2.15 (1.83)
```

These tables show similiar findings to our figures above such
that:

Associated with CDH:

-Decreased (low) Surfactant B

-Increased (high) Osteopontin

-Decreased (low) Kallikrein-5

-Decreased (low) Galectin-3

And that there is no clear time component except that ealy blood
samples had very low levels of kallikrein-5.

# 6 Predication Model - Logistic Regression

Now we�ll start with a classical logistic regression model to predict
disease in the overall cohort (not accounting for time of sample)

```
dat_cdh %>% 
  mutate(disease = if_else(disease == "Control", 0, 1)) -> dat_cdh_log

cdh_modA_glm <- glm(disease ~ surf_b + osteop + kalli_5 + gale_3, data = dat_cdh_log,
                    family = binomial)

glance(cdh_modA_glm)
```

```
# A tibble: 1 � 8
  null.deviance df.null logLik   AIC   BIC deviance df.residual  nobs
          <dbl>   <int>  <dbl> <dbl> <dbl>    <dbl>       <int> <int>
1          55.1      39  -9.01  28.0  36.5     18.0          35    40
```

```
summary(cdh_modA_glm)
```

```
Call:
glm(formula = disease ~ surf_b + osteop + kalli_5 + gale_3, family = binomial, 
    data = dat_cdh_log)

Deviance Residuals: 
    Min       1Q   Median       3Q      Max  
-1.7262  -0.1752   0.0863   0.2395   1.8163  

Coefficients:
            Estimate Std. Error z value Pr(>|z|)   
(Intercept)  15.5250     5.5775   2.784  0.00538 **
surf_b       -0.3997     0.1387  -2.881  0.00396 **
osteop        0.2176     0.1217   1.788  0.07379 . 
kalli_5      -3.1679     2.6050  -1.216  0.22396   
gale_3       -0.1341     0.2640  -0.508  0.61150   
---
Signif. codes:  0 '***' 0.001 '**' 0.01 '*' 0.05 '.' 0.1 ' ' 1

(Dispersion parameter for binomial family taken to be 1)

    Null deviance: 55.051  on 39  degrees of freedom
Residual deviance: 18.019  on 35  degrees of freedom
AIC: 28.019

Number of Fisher Scoring iterations: 8
```

```
confint(cdh_modA_glm, level = 0.95)
```

```
                   2.5 %     97.5 %
(Intercept)   7.14834022 30.1927169
surf_b       -0.77279694 -0.1897884
osteop        0.01382291  0.5289260
kalli_5     -11.00173187 -0.4420814
gale_3       -0.71040690  0.4096274
```

```
cdh_modA <- glm(disease ~ surf_b + osteop + kalli_5 + gale_3, data = dat_cdh_log,
family="binomial"(link="logit"))

tidy(cdh_modA, exponentiate = TRUE, conf.int = TRUE) %>%
select(term, estimate, conf.low, conf.high)
```

```
# A tibble: 5 � 4
  term            estimate     conf.low conf.high
  <chr>              <dbl>        <dbl>     <dbl>
1 (Intercept) 5525996.     1272.         1.30e+13
2 surf_b            0.671     0.462      8.27e- 1
3 osteop            1.24      1.01       1.70e+ 0
4 kalli_5           0.0421    0.0000167  6.43e- 1
5 gale_3            0.875     0.491      1.51e+ 0
```

We see here in our prediction model that the only variable coming up
as significant in our prediction if the `surfactant-b` value.
With a coefficient of -0.40 (P = 0.004). Indicating that as
`surfactant-b` increases the likelihood of CDH decreases.

## 6.1 Visualize Surfactant-B Data

We can see this relationship by looking at the CDH data with respect
to `surfactant_b`

```
dat_cdh %>% 
ggplot(aes(x = disease,y = surf_b)) +
  geom_boxplot(outlier.shape = NA) +
  geom_jitter(aes(col = time), width = 0.15, height = 0.01, size = 0.4) +
  guides() +
  theme_classic() +
  labs(y = "Sufactant-B Levels (mg/dL)")
```

# 7 ROC For Logistic Regression

Now we can assess the accurracy of our model using these 4 ELISA
assays to predict CDH.

```
## requires ROCR package
prob <- predict(cdh_modA_glm, dat_cdh_log, type="response")
pred <- prediction(prob, dat_cdh_log$disease)
perf <- performance(pred, measure = "tpr", x.measure = "fpr")
auc <- performance(pred, measure="auc")
auc <- round(auc@y.values[[1]],3)

roc.data <- data.frame(fpr=unlist(perf@x.values),
tpr=unlist(perf@y.values),
model="GLM")

ggplot(roc.data, aes(x=fpr, ymin=0, ymax=tpr)) +
geom_ribbon(alpha=0.2, fill = "blue") +
geom_line(aes(y=tpr), col = "blue") +
geom_abline(intercept = 0, slope = 1, lty = "dashed") +
labs(title = paste0("ROC Curve w/ AUC=", auc)) +
theme_bw()
```

Our ROC curve for a logistic regression model demonstrates that by
using all 4 ELISA assays we can develop a model with very good accuracy
resulting in an AUC of 0.97 (~97%).

This is a good method and reflets a useful model but does not tell us
about what is contributing to the model and assumes that the
relationship between the covariates and the outcome are linear which may
not be true here.

If we wanted to develop a more simple test we could try using
`surfactant-b` alone as shown below

```
cdh_modA_surf<- glm(disease ~ surf_b, data = dat_cdh_log,
                    family = binomial)

## requires ROCR package
prob <- predict(cdh_modA_surf, dat_cdh_log, type="response")
pred <- prediction(prob, dat_cdh_log$disease)
perf <- performance(pred, measure = "tpr", x.measure = "fpr")
auc <- performance(pred, measure="auc")
auc <- round(auc@y.values[[1]],3)

roc.data <- data.frame(fpr=unlist(perf@x.values),
tpr=unlist(perf@y.values),
model="GLM") 

ggplot(roc.data, aes(x=fpr, ymin=0, ymax=tpr)) +
geom_ribbon(alpha=0.2, fill = "blue") +
geom_line(aes(y=tpr), col = "blue") +
geom_abline(intercept = 0, slope = 1, lty = "dashed") +
labs(title = paste0("ROC Curve w/ AUC=", auc)) +
theme_bw()
```

Here we see that if we use `surfactant-b` alone we drop
our AUC to 0.85. While `surfactant-b` is clearly well
associated with CDH. There is more we can learn more bout this
relationship that may be more clinically applicable.

Therefore, we can use Random Forest to determine more information
about these ELISA assays and our outcome of CDH.

# 8 Random Forest

rfsrc.cdh-binary.R randomSurvivalForest analysis for CDH based on
ELISA Assays CDH prediction; (Apte Lab, n=40)

## 8.1 RF Setting

Random Forest Parameters for Analysis

```
options(object.size=Inf,expressions=100000,memory=Inf,width=130)
options(scipen=3)
event.marks <- c(1,4)
event.labels <- c(FALSE, TRUE)
strCol <- brewer.pal(3, "Set1")
strCol <- strCol[c(2,1,3)]
alpha <- .3
theme_set(theme_bw())


### -----------------------------------------------------------------------------
## Read in the data set, and format it for analysis. 
##
## see tp.dc.graphics.R for a template on this also.
##

# dat_cdh is our dataset, need to make sure binary factor variables are 0 and 1 

### ------------ Data manipulations --------------------------------------------
### ----------------------------------------------------------------------------
## Set modes correctly. For binary variables: transform to logical
## Check for range of 0,1
dat_cdh_log %>%  
  mutate(time = if_else(time == "Early", 0, 1) , 
         disease = if_else(disease == 0, "Control", "CDH"),
         disease = as.factor(disease)) %>% 
  select(-id) -> rf_cdh
```

## 8.2 Start RF - Overall

Run the Random Forest

```
### -------------------RSF -------------------------------------------------------
# Now use that split rule and optimal parameters to analyse the data using all predictors
ntree=1000
set.seed(1)


## Save the forest, so we don't have to rebuild it every time & check error rates. 
(cdh.rfs <- imbalanced(disease ~ ., data = as.data.frame(rf_cdh), na.action="na.imput",
                 block.size=10,
                 ntree=ntree,
                 splitrule="auc", 
                 importance = TRUE,
                 tree.err=TRUE,
                 nsplit=8, 
                 mtry=6, 
                 forest=TRUE, 
                 err.block=TRUE))
```

```
                         Sample size: 40
           Frequency of class labels: 22, 18
                     Number of trees: 1000
           Forest terminal node size: 1
       Average no. of terminal nodes: 6.053
No. of variables tried at each split: 5
              Total no. of variables: 5
       Resampling used to grow trees: swor
    Resample size used to grow trees: 25
                            Analysis: RFQ
                              Family: class
                      Splitting rule: auc *random*
       Number of random split points: 8
                    Imbalanced ratio: 1.2222
                   (OOB) Brier score: 0.14765032
        (OOB) Normalized Brier score: 0.59060127
                           (OOB) AUC: 0.87626263
                        (OOB) PR-AUC: 0.83077471
                        (OOB) G-mean: 0.77849894
   (OOB) Requested performance error: 0.22150106

Confusion matrix:

          predicted
  observed CDH Control class.error
   CDH      16       6      0.2727
   Control   3      15      0.1667

      (OOB) Misclassification rate: 0.225
```

```
save(cdh.rfs, file="rfsrc_cdh.rda")
```

Here we see that the error rate is approximated 22% and that we
missclassify about ~9 patients. We will revisit this later.

## 8.3 RF OOB Error

Error rate stabilizes which is a good sign and demonstrates our model
is stable.

```
### ---------------OOB ERROR: ----------------------------------------------------

err<- cdh.rfs$err.rate

#removed [,1] after err
plot(na.omit(err[,1]), type="l",  xlab="Trees", ylab="Error rate")
```

## 8.4 Variable Selection

Identify variables of importance/interest

```
### --------------variable selection----------------------------------------------

cdh.obj <- max.subtree(cdh.rfs)
print(cdh.obj$topvars)
```

```
[1] "surf_b" "osteop"
```

```
vs.obj <- var.select(cdh.rfs)
```

```
minimal depth variable selection ...


-----------------------------------------------------------
family             : class 
var. selection     : Minimal Depth 
conservativeness   : medium 
x-weighting used?  : TRUE 
dimension          : 5 
sample size        : 40 
ntree              : 1000 
nsplit             : 8 
mtry               : 5 
nodesize           : 1 
refitted forest    : FALSE 
model size         : 2 
depth threshold    : 1.8001 
PE (true OOB)      : 0.2215 NA NA 


Top variables:
       depth vimp.all vimp.CDH vimp.Control
surf_b 0.249    0.161       NA           NA
osteop 1.410    0.035       NA           NA
-----------------------------------------------------------
```

## 8.5 VIMP Plots

Create VIMP dataset

```
### ------------------------------------------------------------------------------
### --------------Variable Importance Plot ---------------------------------------
## 3 column corresponds to binary=True (only noises up true);
## 1 column corresponds to binary=All (noises up all);
cdh.vim <- cdh.rfs$importance

cdh.vimp <- as.data.frame(cbind(cdh.vim[order(cdh.vim, decreasing=TRUE)]))
colnames(cdh.vimp) <-"VIMP"

cdh.vimp$names<-row.names(cdh.vimp)
cdh.vimp$names <- factor(cdh.vimp$names, levels=rev(cdh.vimp$names))

cdh.vimp %>% 
  filter(complete.cases(VIMP)) -> cdh.vimp
```

## 8.6 VIMP Plots (Labelled Figure)

VIMP plot for manuscript

```
###---------- PLOT TOP 5 VARIABLES -------------------------
n.top = 5

cdh.vimp1<- as.data.frame((cdh.vimp[1:n.top,]))

cdh.vimp$vimpcat = !na.omit(as.integer(cdh.vimp$VIMP >=0))

## Manually enter the labels.

cdh.vimp1$names1 <- c("Surfactant-B", "Osteopontin", "Kallikrein-5", "Galectin-3", "Time")
cdh.vimp1$names1 <- factor(cdh.vimp1$names1,
                           levels= c("Time", "Galectin-3", 
                                      "Kallikrein-5", "Osteopontin", "Surfactant-B"))
cdh.vimp1$vimpcat = as.integer(cdh.vimp1$VIMP >=0)


vimp.plt<-ggplot(cdh.vimp1)+
  geom_bar(aes(y=VIMP, x=names1, 
               fill = factor(vimpcat),colour = factor(vimpcat)),
           stat="identity", width=.5) +
  scale_fill_manual(values=c("red", "blue"))+ 
  scale_colour_manual(name="VIMP values",
                      labels=c("Negative","Positive"),
                      values=c("red","blue")) +
  theme(legend.position="none") + 
  labs(x="Variable", y="VIMP") + 
  coord_flip()

pdf("rfsrc-vimp-cdh-top5.pdf", height=9, width=6)
show(vimp.plt) 

dev.off()
```

```
quartz_off_screen 
                2
```

## 8.7 Dependence Plots

```
############################################################################################################
##Variable dependence x-variable and probability of CONTROL
##Individual case predictions are indicated with points (red x indicates stroke, blue - no stroke)
##GAM smooth splines indicates the trend.", echo=FALSE, results=FALSE}

dat_cdh_log %>% 
  select(id) -> dat_id 

gg_v <- gg_variable(cdh.rfs) %>% cbind(dat_id)

gg_v %>% 
  mutate(check = case_when(yhat.CDH > 0.50 & yvar == c("CDH") ~ "Correct", 
                           yhat.CDH > 0.50 & yvar == c("Control") ~ "Incorrect", 
                           yhat.CDH < 0.50 & yvar == c("CDH") ~ "Incorrect", 
                           yhat.CDH < 0.50 & yvar == c("Control") ~ "Correct", 
                           TRUE ~ "Check Again")) -> dat_temp

dat_temp %>% 
  select(id, check) -> dat_incorrect

ggvar<- ggplot(gg_v)+
             geom_point(aes(x=surf_b, y=100*yhat.CDH)) +  
             geom_smooth(aes(x=surf_b, y=100*yhat.CDH), se=FALSE, span=.3, col="brown") +
             scale_colour_manual(name="CDH",
                      labels=c("No","Yes"),
                      values=c("Blue","Red")) +
  theme(
        legend.key = element_blank(),
        panel.grid.major = element_blank(),
        panel.grid.minor = element_blank(),
        legend.title=element_blank(),
        panel.background = element_blank(),
        panel.border = element_blank(),
        axis.line.x = element_line(color="black", size = 0.8),
        axis.line.y = element_line(color="black", size = 0.8),
        axis.text=element_text(size=12, color="black"),
        axis.title=element_text(size=15))+
    labs(y="Probability of CDH (%)", x="Surfactant-B (mg/dL)") 

pdf("rfsrc-variable-dependent-cdh-surfactant_b", height=9, width=6)
show(ggvar)
dev.off()
```

```
quartz_off_screen 
                2
```

## 8.8 Partial Plots

```
########################################################################################
##PARTIAL PLOTS NEW CODE-THE OLD CODE IS AT THE BOTTOM

###OVERALL PLOT
###----------------------------Partial dependence plot-----------------------------------
##Choose the variables that needed partial dependence plot;
## OR for example, can use top 2 or 3 variables from VIMP ;
### -------------------------------------------------------------------------------------
#n.top = 2
#vimp = bdavr.rfs$importance
#key.var = bdavr.rfs$xvar.names[order(vimp, decreasing = TRUE)][1:n.top]

key.var<- c("surf_b", "osteop", "kalli_5", "gale_3", "time")

npts<-40


plt.part <- plot.variable(cdh.rfs, xvar.names=key.var,
                          plots.per.page=1, sorted=FALSE, 
                          show.plots=TRUE, partial=TRUE, 
                          npts=npts, target=2)
```

```
plt.part
```

```
$family
[1] "class"

$partial
[1] TRUE

$event.info
NULL

$target
[1] 2

$ylabel
[1] "probability Control"

$n
[1] 40

$xvar.names
[1] "surf_b"  "osteop"  "kalli_5" "gale_3"  "time"   

$nvar
[1] 5

$plots.per.page
[1] 1

$granule
[1] 5

$smooth.lines
[1] FALSE

$pData
$pData[[1]]
$pData[[1]]$xvar.names
[1] "surf_b"

$pData[[1]]$yhat
 [1] 0.1323761 0.1331207 0.1447045 0.1489973 0.1590833 0.1754259 0.1795229 0.1689930 0.1695383 0.1817176 0.1860489 0.1965779
[13] 0.2073506 0.2517898 0.2513807 0.2665737 0.2934819 0.3417710 0.4236823 0.5976775 0.5930337 0.5993611 0.5953093 0.6601926
[25] 0.6579995 0.6412200 0.6863343 0.6848630 0.6768041 0.7039919 0.7045156 0.6952672 0.7175484 0.7120513 0.7001515 0.7493973
[37] 0.7493973 0.7493973 0.7493973 0.7493973

$pData[[1]]$yhat.se
 [1] 0.01815980 0.01824627 0.01956898 0.02004839 0.02115181 0.02287143 0.02328929 0.02220462 0.02226165 0.02351095 0.02394393
[12] 0.02497172 0.02598702 0.02978734 0.02975521 0.03091319 0.03278496 0.03556986 0.03860755 0.03801992 0.03815995 0.03796747
[23] 0.03809219 0.03547101 0.03558134 0.03637519 0.03403868 0.03412504 0.03458588 0.03294892 0.03291510 0.03349970 0.03204536
[34] 0.03241876 0.03319433 0.02969394 0.02969394 0.02969394 0.02969394 0.02969394

$pData[[1]]$n.x
[1] 40

$pData[[1]]$x.uniq
 [1] 19.56862 25.49964 26.58843 28.27892 29.56827 29.76884 29.79009 33.72285 33.98072 34.20994 35.41334 36.10099 36.64539 36.70009
[15] 36.70269 37.73417 38.04935 39.39601 39.45331 41.45897 42.80005 43.14946 43.46464 44.12001 44.35001 45.61356 46.10065 47.27539
[29] 48.19226 48.73666 48.79396 49.48162 50.42714 50.71367 51.22941 51.60189 52.28003 52.69068 56.87391 62.51842

$pData[[1]]$x
 [1] 42.80005 44.12001 36.70009 29.79009 44.35001 52.28003 41.45897 47.27539 62.51842 48.73666 48.79396 50.42714 52.69068 50.71367
[15] 43.14946 56.87391 51.60189 46.10065 35.41334 33.72285 45.61356 29.56827 36.64539 33.98072 36.70269 36.10099 28.27892 19.56862
[29] 25.49964 26.58843 49.48162 48.19226 43.46464 39.39601 29.76884 34.20994 37.73417 39.45331 51.22941 38.04935


$pData[[2]]
$pData[[2]]$xvar.names
[1] "osteop"

$pData[[2]]$yhat
 [1] 0.6279977 0.6279977 0.6279977 0.6266112 0.6281547 0.6287058 0.6287058 0.6312631 0.6312631 0.6362456 0.6357161 0.6323430
[13] 0.6234664 0.6138881 0.6125974 0.6073569 0.5989721 0.5835767 0.5829878 0.5521531 0.4629776 0.4598424 0.4603084 0.4608260
[25] 0.4614210 0.4427604 0.3872646 0.3863356 0.2966723 0.2966723 0.2964771 0.2964771 0.3186946 0.3209155 0.3219801 0.2966600
[37] 0.2984018 0.3053476 0.3123534 0.3412799

$pData[[2]]$yhat.se
 [1] 0.03693802 0.03693802 0.03693802 0.03699384 0.03693167 0.03690929 0.03690929 0.03680417 0.03680417 0.03659342 0.03661619
[12] 0.03675916 0.03711819 0.03747765 0.03752388 0.03770613 0.03797967 0.03842403 0.03843954 0.03909841 0.03931175 0.03927349
[23] 0.03927937 0.03928583 0.03929314 0.03901043 0.03751896 0.03748570 0.03299170 0.03299170 0.03297915 0.03297915 0.03433101
[34] 0.03445756 0.03451767 0.03299091 0.03310243 0.03353760 0.03396109 0.03554525

$pData[[2]]$n.x
[1] 40

$pData[[2]]$x.uniq
 [1]  0.006427  0.225440  3.097709  3.438602  3.748505  3.973184  4.771184  6.398175  6.537631  6.878524  6.901767  6.917262
[13]  6.932757  7.459592  7.591301  7.815981  8.311826  8.366059 10.814290 10.922760 11.875710 12.363810 13.216040 13.704140
[25] 13.804860 14.424660 14.781050 15.129690 15.656530 17.213790 17.872330 18.461150 18.833030 20.514250 20.831900 22.272950
[37] 22.799790 22.861770 23.737240 24.690200

$pData[[2]]$x
 [1]  3.097709  6.398175  6.917262  6.901767  0.006427  8.311826 10.922760 14.424660 10.814290  7.591301 24.690200 13.804860
[13] 20.831900 15.129690  6.878524  3.973184  7.815981  6.932757 11.875710 17.872330 22.272950  3.748505  7.459592  3.438602
[25] 14.781050 15.656530  8.366059  4.771184  6.537631  0.225440 22.799790 12.363810 23.737240 18.833030 13.216040 13.704140
[37] 20.514250 22.861770 18.461150 17.213790


$pData[[3]]
$pData[[3]]$xvar.names
[1] "kalli_5"

$pData[[3]]$yhat
 [1] 0.4240612 0.4225445 0.4276277 0.4322712 0.4266296 0.4258939 0.4255560 0.4196642 0.4196642 0.4196642 0.4162978 0.4148952
[13] 0.4148952 0.3885312 0.3849951 0.3849951 0.3853990 0.3853990 0.3853990 0.3853990 0.3853990 0.3877187 0.3931244 0.4088936
[25] 0.4476484 0.5137930 0.5137930 0.6522170 0.6522170 0.6522170 0.6522170 0.6522170

$pData[[3]]$yhat.se
 [1] 0.03861667 0.03857989 0.03870031 0.03880317 0.03867731 0.03866015 0.03865222 0.03850803 0.03850803 0.03850803 0.03842072
[12] 0.03838328 0.03838328 0.03756386 0.03743724 0.03743724 0.03745190 0.03745190 0.03745190 0.03745190 0.03745190 0.03753512
[23] 0.03772243 0.03821607 0.03909513 0.03949839 0.03949839 0.03586497 0.03586497 0.03586497 0.03586497 0.03586497

$pData[[3]]$n.x
[1] 32

$pData[[3]]$x.uniq
 [1] 0.000104 0.000134 0.000188 0.000198 0.000263 0.000307 0.000317 0.000351 0.000446 0.000510 0.000575 0.000629 0.000693 0.000822
[15] 0.000866 0.000876 0.000886 0.000941 0.005134 0.010778 0.177442 0.231205 0.233893 0.440879 0.653242 0.798401 0.924743 1.190868
[29] 2.311819 3.430083 3.997279 4.553722

$pData[[3]]$x
 [1] 3.430083 4.553722 1.190868 3.997279 0.798401 2.311819 0.000198 0.000510 0.000263 0.000629 0.000198 0.000104 0.000693 0.000317
[15] 0.000886 0.000822 0.000575 0.000886 0.000866 0.177442 0.000188 0.233893 0.231205 0.440879 0.000876 0.000351 0.653242 0.010778
[29] 0.924743 0.000307 0.000510 0.005134 0.000134 0.000693 0.000307 0.000941 0.000575 0.000446 0.000876 0.000134


$pData[[4]]
$pData[[4]]$xvar.names
[1] "gale_3"

$pData[[4]]$yhat
 [1] 0.4485128 0.4485128 0.4485128 0.4485128 0.4490484 0.4490484 0.4505952 0.4534808 0.4528919 0.4540361 0.4526237 0.4463158
[13] 0.4451865 0.4440575 0.4498544 0.4498544 0.4508554 0.4529180 0.4537891 0.4552483 0.4523035 0.4406372 0.4437724 0.4605645
[25] 0.4706937 0.4810234 0.4841973 0.4923128 0.5140198 0.5177685 0.5261718 0.5267104 0.5267104 0.5273686 0.5273686 0.5273686
[37] 0.5273686 0.5273686 0.5273686 0.5273686

$pData[[4]]$yhat.se
 [1] 0.03910932 0.03910932 0.03910932 0.03910932 0.03911800 0.03911800 0.03914254 0.03918631 0.03917759 0.03919443 0.03917358
[12] 0.03907279 0.03905341 0.03903364 0.03913088 0.03913088 0.03914660 0.03917798 0.03919083 0.03921181 0.03916877 0.03897129
[23] 0.03902859 0.03928258 0.03939267 0.03947153 0.03948899 0.03951913 0.03949739 0.03947855 0.03942017 0.03941567 0.03941567
[34] 0.03941004 0.03941004 0.03941004 0.03941004 0.03941004 0.03941004 0.03941004

$pData[[4]]$n.x
[1] 40

$pData[[4]]$x.uniq
 [1]  0.000000  0.014093  0.095151  0.154593  0.338323  0.570687  0.603110  0.705782  1.186722  1.343433  1.419086  1.592009
[13]  1.597413  1.640643  1.991891  2.062141  2.597119  2.688984  3.148309  3.175328  3.537384  3.656268  3.715710  3.856209
[25]  4.142612  4.148015  4.396591  4.936973  5.228779  6.455447  6.498677  6.974213  7.055271  7.871247  8.217092  8.557532
[37]  8.800704  8.865550 10.108430 13.026490

$pData[[4]]$x
 [1]  4.148015 10.108430  1.343433  5.228779  7.055271 13.026490  0.705782  3.175328  1.597413  8.800704  1.419086  8.557532
[13]  3.537384  6.498677  8.217092  6.974213  7.871247  0.000000  3.148309  0.014093  0.154593  4.396591  2.062141  1.186722
[25]  2.688984  6.455447  3.856209  0.338323  0.570687  1.991891  1.592009  1.640643  3.656268  0.095151  4.936973  4.142612
[37]  2.597119  8.865550  3.715710  0.603110


$pData[[5]]
$pData[[5]]$xvar.names
[1] "time"

$pData[[5]]$yhat
 [1] 0.5661595 0.5788491 0.5180156 0.4909540 0.5755548 0.5664574 0.4952124 0.4987837 0.5609511 0.5785249 0.4734626 0.5526070
[13] 0.4671813 0.5030140 0.5564713 0.5763831 0.5784683 0.5518249 0.4282816 0.4242635 0.5211100 0.4528735 0.4672461 0.4646606
[25] 0.4317740 0.4425219 0.4839130 0.4296839 0.5283566 0.4306826 0.5356190 0.5638085 0.5316299 0.4473778 0.4414376 0.4323278
[37] 0.4293891 0.4941567 0.5654075 0.4384309 0.4706144 0.4787920 0.4152842 0.3997092 0.4641400 0.4467939 0.3751629 0.3618908
[49] 0.4215327 0.4684332 0.3467819 0.4130769 0.3505506 0.3754005 0.4590735 0.4707076 0.4679018 0.4279660 0.3322409 0.3303687
[61] 0.3694555 0.3614720 0.3721192 0.3694555 0.3371874 0.3524552 0.3930855 0.3341547 0.4357868 0.3350970 0.4121977 0.4626221
[73] 0.3759004 0.3411636 0.3459855 0.3364810 0.3331704 0.3881796 0.4059434 0.3372812

$pData[[5]]$yhat.se
NULL

$pData[[5]]$n.x
[1] 2

$pData[[5]]$x.uniq
[1] 0 1

$pData[[5]]$x
 [1] 1 1 1 1 1 1 0 0 0 0 0 0 0 0 0 0 0 0 1 1 1 1 1 1 1 1 1 1 1 1 1 1 0 0 0 0 0 0 0 0


attr(,"class")
[1] "rfsrc"         "plot.variable" "class"
```

```
#save(plt.part, file="../estimates/bdavr_partial_stroke.rda")

#load("../estimates/rfsrc_surv_lvef_steoke.rda")

### ------------------------------------------------------------------------------
### --------------------- Partial plot for Circulatory time -----------------------------------
 

cdh.cat <- as.data.frame(cbind(plt.part$pData[[1]]$x.uniq, plt.part$pData[[1]]$yhat, plt.part$pData[[1]]$yhat.se))
                           
colnames(cdh.cat)<- c("x", "prob", "se")

cdh.cat %>% mutate(prob = 1 - prob) -> cdh.cat_cor


surf_b.plt <- ggplot(cdh.cat_cor) +
           geom_point(aes(x=x, y=100*prob), col= "red" ) +
           geom_smooth(aes(x=x, y=100*prob), se=FALSE, span=.3, col="black") + 
  # Everything else is just formatting for publication
  # scale_y_continuous(limits=c(20, 90), expand = c(0, 0))+
  # scale_x_continuous(limits=c(15, 70), breaks=seq(15, 70, 10), expand=c(0,0))+
  theme(legend.position="none",
        legend.key = element_blank(),
        panel.grid.major = element_blank(),
        panel.grid.minor = element_blank(),
        legend.title=element_blank(),
        panel.background = element_blank(),
        panel.border = element_blank(),
        axis.line.x = element_line(color="black", size = 0.8),
        axis.line.y = element_line(color="black", size = 0.8),
        axis.text=element_text(size=12, color="black"),
        axis.title=element_text(size=15))+
         labs(x="Surfactant-B (mg/dL)", y="CDH Probability (%)") 

surf_b.plt
```

```
pdf("Partial_surfactact_b_CDH.pdf", height=6,width=8);
surf_b.plt
dev.off()
```

```
quartz_off_screen 
                2
```

## 8.9 Stratify by Time

```
#####STRATIFY BY GROUP VARIABLE- Time

##Surf-B PARTIAL PLOT STRATIFIED BY TIME
##############Subset for Early = 0
subset.early0 <- which(cdh.rfs$xvar$time == 0)
plt.part.early0 <- plot.variable(cdh.rfs, xvar.names=key.var,
                          plots.per.page=1, sorted=FALSE, 
                          show.plots=TRUE, partial=TRUE, 
                          npts=npts, target=2,subset = subset.early0)
```

```
### ------------------------------------------------------------------------------
### --------------------- Partial plot Time (Early) -----------------------------------


cdh.cat.early0 <- as.data.frame(cbind(plt.part.early0$pData[[1]]$x.uniq, plt.part.early0$pData[[1]]$yhat, plt.part.early0$pData[[1]]$yhat.se))

colnames(cdh.cat.early0)<- c("x", "prob", "se")

cdh.cat.early0 %>% 
  mutate(prob = 1 - prob) -> cdh.cat.early0_cor

##############Subset for Term = 1
subset.term1 <- which(cdh.rfs$xvar$time == 1) 
plt.part.term1 <- plot.variable(cdh.rfs, xvar.names=key.var,
                                  plots.per.page=1, sorted=FALSE, 
                                  show.plots=TRUE, partial=TRUE, 
                                  npts=npts,target=2,subset = subset.term1)
```

```
#?Notch = False

### ------------------------------------------------------------------------------
### --------------------- Partial plot Time (Term) -----------------------------------


cdh.cat.term1 <- as.data.frame(cbind(plt.part.term1$pData[[1]]$x.uniq, plt.part.term1$pData[[1]]$yhat, plt.part.term1$pData[[1]]$yhat.se))

colnames(cdh.cat.term1)<- c("x", "prob", "se")


cdh.cat.term1 %>% 
  mutate(prob = 1 - prob) -> cdh.cat.term1_cor

###graph for Time=0 and Time=1

cdh.plt.time <- ggplot(cdh.cat_cor) +
  geom_point(aes(x=x, y=100*prob), col="black") +
  geom_smooth(aes(x=x, y=100*prob), se=FALSE, span=.3, col="black") +  
  
  geom_point(data=cdh.cat.early0_cor,aes(x=x, y=100*prob), col="blue") +
  geom_smooth(data=cdh.cat.early0_cor, aes(x=x, y=100*prob), se=FALSE, span=.3, col="blue") +  
  
  geom_point(data=cdh.cat.term1_cor,aes(x=x, y=100*prob), col="red") +
  geom_smooth(data=cdh.cat.term1_cor, aes(x=x, y=100*prob), se=FALSE, span=.3, col="red") +
  # Everything else is just formatting for publication
  # scale_y_continuous(limits=c(20, 90), expand = c(0, 0))+
  # scale_x_continuous(limits=c(15, 70), breaks=seq(15, 70, 10), expand=c(0,0))+
  theme(legend.position="none",
        legend.key = element_blank(),
        panel.grid.major = element_blank(),
        panel.grid.minor = element_blank(),
        legend.title=element_blank(),
        panel.background = element_blank(),
        panel.border = element_blank(),
        axis.line.x = element_line(color="black", size = 0.8),
        axis.line.y = element_line(color="black", size = 0.8),
        axis.text=element_text(size=12, color="black"),
        axis.title=element_text(size=15))+
         labs(x="Surfactant-B (mg/dL)", y="CDH Probability (%)") 
  
  #Blue = Early, # Red = Term
  
  
pdf("cdh.rfsrc_surf_b.time.pdf",height=7,width=8)
show(cdh.plt.time)
dev.off()
```

```
quartz_off_screen 
                2
```

## 8.10 Checking Incorrect Assignments

Now we will check the incorrect cases to try and indentify why there
were misclassified by our model.

```
dat_incorrect <- left_join(dat_cdh, dat_incorrect)

#Boxplot of Surf_B
dat_incorrect %>% 
ggplot(aes(x = check,y = surf_b)) +
  geom_point(aes(color = disease)) +
  guides() +
  theme_classic() +
  labs(y = "Sufactant-B Levels (mg/dL)")
```

```
#Boxplot of Osteopontin
dat_incorrect %>% 
ggplot(aes(x = check,y = osteop)) +
  geom_point(aes(color = disease)) +
  guides() +
  theme_classic() +
  labs(y = "Osteopontin Levels (mg/dL)")
```

```
#Boxplot of Kallikrein-5
dat_incorrect %>% 
ggplot(aes(x = check,y = kalli_5)) +
  geom_point(aes(color = disease)) +
  guides() +
  theme_classic() +
  labs(y = "Kallikrein-5 Levels (mg/dL)")
```

```
#Boxplot of Galectin-3
dat_incorrect %>% 
ggplot(aes(x = check,y = gale_3)) +
  geom_point(aes(color = disease)) +
  guides() +
  theme_classic() +
  labs(y = "Galectin-3 Levels (mg/dL)")
```

Based on this curves it appears like most of the incorrectly assigned
cases were from those with average `surfactant-b` levels as
well as elevated `osteopontin` levels. While out model�s out
of bag error was ~22% that is still relatively strong given the small
sample size.

# 9 Conclusion

Based on these analysis we demonstrate that the use of
`surfactant-b`, `ostepontin`,
`kallikrein-5`, and `galectin-3` protein levels
can differentiate mothers with CDH infants from Control mothers without
CDH infants. Based on our logistic regression and Random Forest we see
that the most influential predictor of CDH is `surfactant-b`,
and that there appears to be a cutoff point near 36 mg/dL where the
probability of CDH shows a non-linear decrease, independent of time of
sample collection. Our models performed well, despite the small sample
size, with an AUC of 0.97 (logistic regression) and an RF OOB (Out of
bag) Error of 0.22.

# 10 Manuscript Figures

## 10.1 Variable of Important (VIMP) Plot

```
vimp.plt<-ggplot(cdh.vimp1)+
  geom_bar(aes(y=VIMP, x=names1, 
               fill = factor(vimpcat),colour = factor(vimpcat)),
           stat="identity", width=.5) +
  scale_fill_manual(values=c("red", "blue"))+ 
  scale_colour_manual(name="VIMP values",
                      labels=c("Negative","Positive"),
                      values=c("red","blue")) +
  theme(legend.position="none") + 
  labs(x="Variable", y="VIMP") + 
  coord_flip()


ggsave("manuscript_vimp.pdf", height=9, width=6)

show(vimp.plt)
```

## 10.2 Surfactant B Partial Plot

```
cdh.plt.time <- ggplot(cdh.cat_cor) +
  geom_point(aes(x=x, y=100*prob), col="black") +
  geom_smooth(aes(x=x, y=100*prob), se=FALSE, span=.3, col="black") +  
  
  geom_point(data=cdh.cat.early0_cor,aes(x=x, y=100*prob), col="blue") +
  geom_smooth(data=cdh.cat.early0_cor, aes(x=x, y=100*prob), se=FALSE, span=.3, col="blue") +  
  
  geom_point(data=cdh.cat.term1_cor,aes(x=x, y=100*prob), col="red") +
  geom_smooth(data=cdh.cat.term1_cor, aes(x=x, y=100*prob), se=FALSE, span=.3, col="red") +
  # Everything else is just formatting for publication
  # scale_y_continuous(limits=c(20, 90), expand = c(0, 0))+
  # scale_x_continuous(limits=c(15, 70), breaks=seq(15, 70, 10), expand=c(0,0))+
  theme(legend.position="none",
        legend.key = element_blank(),
        panel.grid.major = element_blank(),
        panel.grid.minor = element_blank(),
        legend.title=element_blank(),
        panel.background = element_blank(),
        panel.border = element_blank(),
        axis.line.x = element_line(color="black", size = 0.8),
        axis.line.y = element_line(color="black", size = 0.8),
        axis.text=element_text(size=12, color="black"),
        axis.title=element_text(size=15))+
         labs(x="Surfactant-B (mg/dL)", y="CDH Probability (%)") 
  
  #Blue = Early, # Red = Term
  
ggsave("manuscript_surf_b_partial_plot.pdf",height=7,width=8)
```

```
`geom_smooth()` using method = 'loess' and formula 'y ~ x'
`geom_smooth()` using method = 'loess' and formula 'y ~ x'
`geom_smooth()` using method = 'loess' and formula 'y ~ x'
```

```
show(cdh.plt.time)
```

```
`geom_smooth()` using method = 'loess' and formula 'y ~ x'
`geom_smooth()` using method = 'loess' and formula 'y ~ x'
`geom_smooth()` using method = 'loess' and formula 'y ~ x'
```
